# The Xylulose 5-Phosphate/Phosphate Translocator Supports Triose Phosphate, but Not Phosphoenolpyruvate Transport Across the Inner Envelope Membrane of Plastids in *Arabidopsis thaliana* Mutant Plants

**DOI:** 10.3389/fpls.2018.01461

**Published:** 2018-10-18

**Authors:** Elke J. A. Hilgers, Pia Staehr, Ulf-Ingo Flügge, Rainer E. Häusler

**Affiliations:** ^1^Department of Biology, Botany II and Cluster of Excellence on Plant Sciences (CEPLAS), Cologne Biocenter, University of Cologne, Cologne, Germany; ^2^Lophius Biosciences GmbH, Regensburg, Germany

**Keywords:** phosphate translocators, metabolite transport, phosphoenolpyruvate, kinetics, mutants

## Abstract

The xylulose 5-phosphate/phosphate translocator (PTs) (XPT) represents a link between the plastidial and extraplastidial branches of the oxidative pentose phosphate pathway. Its role is to retrieve pentose phosphates from the extraplastidial space and to make them available to the plastids. However, the XPT transports also triose phosphates and to a lesser extent phosphoenolpyruvate (PEP). Thus, it might support both the triose phosphate/PT (TPT) in the export of photoassimilates from illuminated chloroplasts and the PEP/PT (PPT) in the import of PEP into green or non-green plastids. In mutants defective in the day- and night-path of photoassimilate export from the chloroplasts (i.e., knockout of the TPT [*tpt-2*] in a starch-free background [*adg1-1*])the XPT provides a bypass for triose phosphate export and thereby guarantees survival of the *adg1-1/tpt-2* double mutant. Here we show that the additional knockout of the XPT in *adg1-1/tpt-2/xpt-1* triple mutants results in lethality when the plants were grown in soil. Thus the XPT can functionally support the TPT. The PEP transport capacity of the XPT has been revisited here with a protein heterologously expressed in yeast. PEP transport rates in the proteoliposome system were increased with decreasing pH-values below 7.0. Moreover, PEP transport determined in leaf extracts from wild-type plants showed a similar pH-response, suggesting that in both cases PEP^2-^ is the transported charge-species. Hence, PEP import into illuminated chloroplasts might be unidirectional because of the alkaline pH of the stroma. Here the consequence of a block in PEP transport across the envelope was analyzed in triple mutants defective in both PPTs and the XPT. PPT1 is knocked out in the *cue1* mutant. For PPT2 two new mutant alleles were isolated and established as homozygous lines. In contrast to the strong phenotype of *cue1*, both *ppt2* alleles showed only slight growth retardation. As plastidial PEP is required e.g., for the shikimate pathway of aromatic amino acid synthesis, a block in PEP import should result in a lethal phenotype. However, the *cue1-6/ppt2-1/ppt2-1* triple mutant was viable and even exhibited residual PEP transport capacity. Hence, alternative ways of PEP transport must exist and are discussed.

## Introduction

In *Arabidopsis thaliana* the family of phosphate translocators (PTs) of the plastidial inner envelope membrane consists of four functional classes with a total of six members. The triose phosphate *(TP)/PT* (*TPT*; Fliege et al., 1978; Flügge et al., 1989) and the xylulose 5-phosphate *(Xu5P)/PT* (*XPT*; Eicks et al., 2002) represent single copy genes, whereas both the glucose 6-phosphate *(Glc6P)/PT* (*GPT*; Kammerer et al., 1998) and the phosphoenolpyruvate *(PEP)/PT* (*PPT*, Fischer et al., 1997) are encoded by two genes each, resulting in the respective GPT1[2] (i.e., GPT1 and GPT2) and PPT1[2] proteins.

The large variations in temporal and spatial expression pattern underpin the functional diversity of the different members of the PT family (e.g., electronic fluorescent protein browser; *efp* browser^1^, Winter et al., 2007).

The TPT is highly abundant in photosynthetic tissues and mediates the export of TPs from the chloroplast stroma in the light to support sucrose biosynthesis in the cytosol. Thereby it provides the whole plant with carbohydrates during the day (‘day path of carbon’). The XPT is ubiquitously expressed in *A. thaliana* and is proposed to serve as a link between the plastidial and extraplastidial branches of the oxidative pentose phosphate pathway (OPPP; Eicks et al., 2002; Kruger and von Schaewen, 2003; Meyer et al., 2011; Hölscher et al., 2016). Indeed, Hilgers et al. (2018) could recently deliver experimental evidence for this role. The GPTs are mainly expressed in heterotrophic tissues, in particular during gametophyte and embryo development (Niewiadomski et al., 2005), and provide Glc6P as a substrate for starch synthesis and/or the OPPP. Finally, the PPTs are expressed in both green and non-green tissues and are proposed to supply plastids lacking a complete glycolysis with PEP as a substrate for the shikimate pathway, which is entirely localized in the plastid stroma and from which aromatic amino acids and a plethora of primary and secondary plant products derive (Tzin and Galili, 2010).

The individual members of the PT family possess both distinct and overlapping specificities for their transport substrates as is summarized in **Table 1**. For instance, the TPT, GPTs, and XPT share TP and 3-PGA as a common substrate albeit with different transport capacities and substrate binding affinities. Both GPTs and to a lesser extent the XPT might also be capable of transporting PEP. Hence PTs that share a common substrate might support each other provided that their temporal and spatial expression patterns overlap.

**Table 1:** Reported transport characteristics for PTs from different plants species (i.e., *Spinacia oleracea* [*So*], *Brassica oleracea* var. *cauli* [*Bo*], *Arabidopsis thaliana* [*At*], and *Pisum sativum* [*Ps*]).

Transport substrate	*So*TPT		*Bo*PPT		*At*PPT1	*At*PPT2	*Ps*GPT		*At*GPT1	*At*GPT2	*At*XPT	
								
	*V*_max_	*K*_m_; *K*_i_	*V*_max_	*K*_m_; *K*_i_	*V*_max_	*V*_max_	*V*_max_	*K*_m_; *K*_i_	*V*_max_	*V*_max_	*V*_max_	*K*_m_; *K*_i_
	(% of P_i_)	(mM)	(% of P_i_)	(mM)	(% of P_i_)	(% of P_i_)	(% of P_i_)	(mM)	(% of P_i_)	(% of P_i_)	(% of P_i_)	(mM)
Phosphate (P_i_)	100^a^	1.0 ± 0.3^a^	100^a^	0.8 ± 0.1^a^	100^b^	100^b^	100^c^	1.1 ± 0.1^c^	100^d^	100^d^	100^e^	1.0 ± 0.2^e^
Triose phosphates	92 ± 17^a^	1.0 ± 0.1^a^	22 ± 3^a^	8.0 ± 0.1^a^	N.D.	N.D.	116 ± 5^c^	0.6 ± 0.1^c^	48 ± 14^d^	106 ± 17^d^	88 ± 12^e^	0.4 ± 0.1^e^
3-PGA	90 ± 16^a^	1.0 ± 0.2^a^	16 ± 3^a^	4.6 ± 0.8^a^	20 ± 3^b^	26 ± 5^b^	50 ± 1^c^	1.8 ± 0.1^c^	88 ± 9^d^	61 ± 1^d^	69 ± 10^e^	10.6 ± 2.9^e^
2-PGA	4 ± 1^a^	12.6 ± 2.7^a^	72 ± 1^a^	5.7 ± 0.1^a^	42 ± 3^b^	32 ± 1^b^	N.D.	N.D.	N.D.	N.D.	N.D.	N.D
PEP	5 ± 2^a^	3.3 ± 0.3^a^	72 ± 9^a^	0.3 ± 0.1^a^	49 ± 5^b^	81 ± 1^b^	20 ± 1^c^	2.9 ± 0.2^c^	35 ± 10^d^	41 ± 4^d^	10 ± 1^e^	2.1 ± 0.2^e^
Glc6P	5 ± 4^a^	>50^a^	2 ± 1^a^	>50a	N.D.	N.D.	90 ± 9^c^	1.1 ± 0.1^c^	116 ± 20^d^	103 ± 5^d^	6 ± 2^e^	66 ± 7^e^
Glc1P	N.D.	N.D.	4 ± 5^a^	N.D.	N.D.	N.D.	<1^c^	N.D.	N.D.	N.D.	<1^e^	N.D
Fru6P	N.D.	N.D.	N.D.	N.D.	N.D.	N.D.	<1^c^	N.D.	N.D.	N.D.	<1^e^	N.D
Xu5P	N.D.	N.D.	N.D.	N.D.	N.D.	N.D.	54 ± 12^e^	N.D.	N.D.	N.D.	81 ± 8^e^	0.8 ± 0.2^e^
Ri5P	N.D.	N.D.	N.D.	N.D.	N.D.	N.D.	29 ± 0^e^	N.D.	N.D.	N.D.	2 ± 1^e^	22 ± 1^e^
Ru5P	N.D.	N.D.	N.D.	N.D.	N.D.	N.D.	53 ± 6^e^	N.D.	N.D.	N.D.	28 ± 4^e^	3.5 ± 0.3^e^
Ery4P	N.D.	N.D.	N.D.	N.D.	N.D.	N.D.	28 ± 8^e^	N.D.	N.D.	N.D.	34 ± 5^e^	3.3 ± 0.5^e^


*The data were compiled from Fischer et al. (1997)^*a*^, Knappe et al. (2003)^*b*^, Kammerer et al. (1998)^*c*^, Niewiadomski et al. (2005)^*d*^, and Eicks et al. (2002)^*e*^.*

Here we ask the question whether the XPT, because of its ubiquitous spatial and temporal expression pattern, might be able to support the TPT and the PPTs, which are expressed in a distinct way. In order to address this question we took advantage of already existing and also of new *A. thaliana* mutant lines. Some of these lines have already played a predominant role in the functional characterization of the transporters, like (1) mutants of the TPT, (2) PPT1, or (3) XPT.

(1) A knockout of the TPT (i.e., a loss of the ‘day path of carbon’) can be almost completely compensated for by an increased turnover of transitory starch. Hence, sucrose biosynthesis starts from the major starch degradation products, maltose and glucose (Zeeman et al., 2010). This ‘night path of carbon’ can be switched on even during the day as has been shown for antisense *TPT* plants of tobacco (Häusler et al., 1998) and different alleles of *A. thaliana* TPT mutants (Schneider et al., 2002; Walters et al., 2004; Schmitz et al., 2012). The survival of the TPT mutants (i.e., *tpt-1* and *tpt-2*) was challenged by crossing them to starch-free mutants (e.g., *adg1-1*, defective in the catalytic subunit of ADP-Glucose pyrophosphorylase; Lin et al., 1988). The resulting double mutant alleles *adg1-1/tpt-1* (Schneider et al., 2002) and *adg1-1/tpt-2* (Schmitz et al., 2012) were severely retarded in growth, exhibited a high chlorophyll fluorescence phenotype in the dark-adapted state and impaired photosynthesis (Häusler et al., 2009; Schmitz et al., 2012). It has been assumed, but never demonstrated experimentally, that the XPT guarantees survival of these double mutants.
(2) For both PPTs so far only PPT1 mutant alleles are available. The *chlorophyll* a/b *binding protein underexpressed1* (*cue1*) mutant of *A. thaliana*, defective in PPT1, (Li et al., 1995; Streatfield et al., 1999), exhibits a strong developmental phenotype characterized by growth retardation of shoot and roots as well as reticulate leaves. An additional knockout of the plastidial enolase (*eno1*), which had previously been functionally characterized (Prabhakar et al., 2009), in the background of the *cue1* mutant resulted in lethality of the double homozygous mutants (Prabhakar et al., 2010). Obviously import and glycolysis as two major ways to provide plastidial PEP were blocked simultaneously in a critical developmental state of the embryo, and resulted in its abortion. Even heterozygous *eno1* mutants in the homozygous *cue1* background showed more severe growth retardation compared to *cue1*, combined with structural changes in both sporopollenin of the male gametophyte and cuticle wax composition on the epidermis of vegetative tissues (Prabhakar et al., 2010). These analyses supported the notion that imported PEP is not only required for the shikimate pathway, but can also be fed into e.g., *de novo* fatty acid biosynthesis (e.g., Thelen and Ohlrogge, 2002) and the methylerythritol 4-phosphate (MEP) pathway of plastidial isoprenoid biosynthesis (Lichtenthaler, 1999) after conversion to pyruvate by plastidial pyruvate kinase.
(3) Surprisingly, loss of function mutants of the XPT lacked any visible or biochemical phenotype, whereas double mutants defective in both the XPT and TPT were retarded in growth and impaired in photosynthesis (Hilgers et al., 2018) and thereby resembled TPT mutants in the starch-free background (e.g., *adg1-1/tpt-2*).

In the first part of this report we could experimentally verify that the XPT can support the TPT. Plants with a complete block of photoassimilate export from the chloroplasts were not able to survive when grown in soil. In the second part, we could confirm that the XPT is capable of transporting PEP, and for the third part, i.e., the block of PEP import into chloroplasts; we isolated two new PPT2 mutant alleles and generated triple mutants defective in PPT1, PPT2 and the XPT. Surprisingly, the triple mutants lacked any additive phenotype to *cue1* and, most strikingly, it exhibited a substantial PEP transport activity. The physiological consequences going along with these observations are discussed.

## Materials and Methods

### Plant Material and Growth Conditions

Seeds of *A. thaliana* ecotypes Ws-2 and Col-0 were obtained from the Nottingham Arabidopsis Stock Centre (NASC). As outlined by Hilgers et al. (2018) the *xpt-1* mutant allele (Ws-2 background) was isolated from the T-DNA insertion pool P39F3 obtained from the Arabidopsis Knockout Facility (Madison, Wisconsin, United States). The pool confers BASTA resistance as a selection marker. In addition, the following mutant lines defective in the gene indicated were used for crossings: *gpt2-1* (At1g61800; Niewiadomski et al., 2005), *adg1-1*/*tpt-2* (Schmitz et al., 2012). The latter double mutant was obtained from crosses between *adg1-1* (At5g48300; Lin et al., 1988) and the *tpt-2* allele (At5g46110; Schmitz et al., 2012). Furthermore the *PPT1* (At5g33320) knockout mutant *cue1-6* (Li et al., 1995) was used in the present study. The isolation of new *PPT2* (At3g01550) knockout mutant alleles is described below. Apart from *xpt-1* all other mutant lines were in the Col-0 background.

In order to identify mutants in the *PPT2* gene we took advantage of the GABI-TILLING (Targeting Induced Local Lesions in Genomes) approach, which had been set up at the University of Potsdam^2^. This approach is based on the creation of mutant populations by EMS and the setup of DNA pools of these mutagenized lines. For the identification of *PPT2* mutants, the DNA pools were screened for base pair exchanges by heteroduplex formation in the *PPT2* gene. The mutagenized Arabidopsis lines were provided by Thomas Altmann and Georg Strompen. The most promising mutant line was a splice variant at position 879 of the *PPT2* gene leading to a frame shift and a series of translational stop codons thereafter. The homozygous mutant line was named *ppt2-1*. As second *PPT2* mutant the T-DNA insertion line Salk_208205 was obtained from the Nottingham stock center (NASC ID: N698426). The homozygous mutant line was named *ppt2-2*.

If not stated otherwise, plants were germinated and grown in soil (Einheitserde, Type Minitray, Gebr. Patzer KG, Sinntal-Jossa) mixed with 30% Vermiculite for approximately 2–3 weeks in a growth chamber (Johnson Control) equipped with eight dimmable fluorescence tubes (Osram L18W/840) in the long-day (16 h light/8 h dark) at a photon flux densities (PFD) at rosette leaf level of 150 μmol⋅m^-2^⋅s^-1^. The humidity was kept at approximately 60% at day/night temperature of 21°C/18°C. For growth studies time point zero refers to ‘days after sowing’ (DAS) excluding a 48 h period of stratification at 4°C.

### Crossing and Propagation of Mutant Plants

For crossings immature flowers of homozygous single mutants were emasculated and manually cross-pollinated. For double mutants, the mutant mentioned first was used as female parent. The *xpt-1* mutant line was crossed to *adg1-1* to obtain *adg1-1/xpt-1* double mutants. These were in turn crossed to the *adg1-1/tpt-2/gpt2-1* triple mutant (Schmitz et al., 2012) with the aim to generate *adg1-1/tpt-2/xpt-1/gpt2-1* quadruple mutants. Homozygous mutants were either verified by PCR on genomic DNA (i.e., for the T-DNA insertion mutants *xpt-1* and *tpt-2*; **Supplementary Table S1**) or by iodine staining of starch in leaves toward the end of the light period (i.e., for *adg1-1*, which was obtained by mutagenesis with ethyl methanesulfonate [EMS]; Lin et al., 1988).

Because of the expected serious growth retardations of plants with a combined homozygous loss of ADG1, TPT and XPT functions, the progenies of the above crosses were grown on 1/2 MS agar plates supplemented with modified vitamins (Duchefa M0245) and 50 mM sucrose as carbon source. Propagation of homozygous triple or quadruple mutants (with the additional loss of GPT2 function) in sterile culture was possible only in one case, in particular humidity and condensation had to be controlled carefully during flowering and silique development. Jelly jars with a lid or Magenta boxes with an air filter were not suitable for propagation. Hence the plants were grown on agar (see above) in open jelly jars that were placed in Microboxes (model type: TP4000 + TPD4000, Combiness Europe, Nevele, Belgium) equipped with an air filter (XXL+: *green filter*) in the lid. The boxes were positioned in a temperature controlled growth cabinet that was run under long-day conditions at day/night temperature of 21°C/18°C. The PFD at plant level was approximately 60 μmol⋅m^-2^⋅s^-1^. The mutant plants were transferred to fresh medium every two weeks.

The *cue1-6* mutant was crossed to *ppt2-1* or *xpt-1* yielding the viable homozygous double mutants *cue1-6/ppt2-1* or *cue1-6/xpt-1*. Both double mutants were again crossed to obtain the *cue1-6/ppt2-1/xpt-1* triple mutant. The progenies of the crosses were screened by PCR on genomic DNA (i.e., *xpt-1, tpt-2, ppt2-1*, **Supplementary Table S1**). The homozygous knockout of *PPT1* in the *cue1-6* mutant (an EMS mutant) in double or triple mutants was verified by its reticulate leaf phenotype and the harsh growth retardation, which were absent in the heterozygous state (Prabhakar et al., 2010).

### DNA and RNA Extraction and PCR Methods

Genomic DNA was isolated according to Edwards et al. (1991). RNA was extracted in hot phenol as described in Collart and Oliviero (2001). After treatment with RNase-free DNase (Ambion), oligo-(dT)-primed cDNA of total RNA was synthesized using the Bioscript reverse transcriptase (Bioline). PCR with cDNA or gDNA was performed using *Taq* polymerase (Thermo Fisher Scientific) according to Mullis et al. (1986) at between 26 and 32 cycles. Amplified DNA was separated on 1.0% agarose gels in the presence of Tris-acetate buffer and 5 μg⋅ml^-1^ ethidium bromide.

### Determination of the Area Growth of Leaf Rosettes

The area growth of leaf rosettes was determined non-destructively from pictures taken between days 11 and 21 after sowing of plants grown in soil or on 1/2MS agar plates. The areas were calculated using ImageJ^3^ by comparing with a known area. The areas were not corrected for overlapping leaves (occurring at later stages of development). Shaded leaves contribute less to photosynthesis as compared to leaves exposed to light. In some cases rosettes were harvested at the same time points and the fresh weights determined.

### Pigment and Protein Determinations

Photosynthesis-associated pigments were extracted from freshly harvested leaf material in ice cold 100% methanol overnight at 4°C in the dark. Chl *a*, *b*, and carotenoid contents were determined as described in Wellburn and Lichtenthaler (1984) at wavelengths of 470, 653, and 666 nm.

Proteins were extracted from frozen leaf material in a medium containing 50 mM Hepes-KOH (pH 7.5), and 0.1% (v/v) Triton-X-100 with a Heidolph homogenizer and determined according to Bradford (1976) using BSA as a standard.

### Heterologous Expression of the XPT cDNA in Baker’s Yeast, and Isolation of the Recombinant Protein

For the analysis of transport characteristics in the proteoliposome system, the *XPT* cDNA was heterologously expressed in *Saccharomyces cerevisiae* and the gene product isolated. In contrast to Eicks et al. (2002), the Gateway System (Invitrogen) was used in the present study.

The ENTRY vector required for the Gateway system was generated by TOPO cloning according to the manufacturers’ guidelines (Invitrogen). The forward primer for cloning of the full length *XPT* cDNA (**Supplementary Table S1**) contained the motif CACC at the 5′ end, which is essential for the TOPO reaction. Following the ligation of the gene fragment into the linearized pENTRY/D-TOPO vector by the topoisomerase reaction, chemically competent *E. coli* (DH5α) cells were transformed and the plasmid DNA was isolated from positive-tested clones. Subsequently the construct was transferred *via* Gateway cloning to the binary yeast expression vector pYES-DEST52 under control of the *Gal* promoter. For both, the immunological identification of the gene products and the purification of the protein by Nickel NTA chromatography (Bornhorst and Falke, 2000), the pYES-DEST52 vector also allowed the translational fusion of the *XPT* cDNA with a hexahistidin tag (His-tag).

Competent yeast cells (*S. cerevisiae*; strain InvSC) were transformed with the pYES-DEST52 vector containing the *XPT* cDNA according standard transformation protocols (Sigma Aldrich^4^). Positive yeast clones were selected on YNB-U + Glc plates and tested for the construct by colony PCR (for primers see **Supplementary Table S1**).

For expression of the recombinant protein, yeast cells tested positive for the construct were incubated under shaking (200 rpm) in 300 ml YNB-U + Glc medium at 28°C for about 38 h. From these precultured cells, aliquots were spun down for 5 min at 20100⋅g and the sediments resuspended in and diluted with a total of 2 l induction medium (YNB-U + Gal). Care was taken that the OD_600_ was close to 0.4 before initiating the induction of *XPT* expression. The cultures were further incubated at 28°C and 5 ml samples were taken and frozen in liquid N_2_ in a time-course (i.e., 0, 2, 4, 6, and 8 h after induction as well as overnight) to monitor the expression strength of the recombinant protein on immunoblots after SDS–PAGE.

In order to yield recombinant proteins at their maximum expression levels from the induced yeast cells, a 1 l culture was distributed equally between three Sorvall GS3 tubes and centrifuged at 11950⋅g for 13 min. Centrifugations and all further steps were done at 4°C. The supernatants were discarded, the sediments resuspended in about 1–2 ml induction medium, collected in 50 ml Falcon tubes and spun down at 20100⋅g for 5 min. The sediments were either directly used for further experiments or frozen at -20°C until use.

For the isolation of yeast membranes the sedimented yeast cells were resuspended in 5 ml ice-cold extraction buffer containing 20 mM Tris–HCl (pH 8.0), 500 mM NaCl, 1 mM DTT, 0.5 mg⋅ml^-1^ PMSF, a few mg of both DNAse and RNAse. Of the suspension 500 μl aliquots were added to 400 mg of acid-washed glass beads in 1.5 ml Eppendorf tubes. The yeast cells were extracted with the glass beads in a MSK cell homogenizer (Braun, Melsungen) for 10 min at 4°C. To the extracts another 500 μl of extraction buffer was added, shaken and centrifuged at 9280⋅g for 5 min. The supernatants were unified in a 15 ml Falcon tube and divided equally into 10 Eppendorf tubes (2 ml). The extracts were centrifuged at 35000 g for 30 min in an Optima L-80 XP Ultracentrifuge. The supernatants were discarded and the sediments containing the yeast membranes stored at -80°C until further use.

### Transport Measurements of Phosphate Translocators in the Reconstituted Proteoliposome System

Transport rates of the heterologously expressed recombinant XPT protein were determined essentially as described by Flügge (1992) and Fischer et al. (1994) in the proteoliposome system.

Aliquots of the frozen yeast membranes containing the recombinant XPT protein were emulsified in 300 μl extraction medium containing 200 mM Tricine-KOH (pH 8.0), 10 mM EDTA, 4 mM DTT, 6 mM ascorbate, and 0.1% w/v defatted BSA. In order to obtain homogenous yeast membrane preparation, the emulsion was sonicated with 5 pulses at 30% duty cycle, output control 5 (Branson Sonifier 250; Branson, MO, United States). For each transport measurement 50 μl of yeast membranes was used.

Liposomes were prepared from purified phospholipids (20% final concentration: Type IV-S, Sigma-Aldrich), which were emulsified in liposome medium containing (final concentrations, each), 100 mM Tricine-KOH (pH 7.8), 50 mM K-gluconate, supplemented with 30 mM of the transport substrates P_i_, or PEP, or as a control with Na-gluconate. Each assay mixture (i.e., for 5 to 10 assays) was sonicated for 10 min at 30% duty cycle, output control 5. Of the extraction medium, containing 50 μl of yeast membranes, 0.6 ml were mixed with 0.5 ml of the desired liposome/substrate preparation, frozen in liquid nitrogen and stored at -80°C until further use. Likewise, for the determination of PT transport activities in wild-type and mutant plants, rosette leaves were pulverized in liquid nitrogen and homogenized in extraction medium supplemented with 0.1% (w/v) of Polyclar AT (insoluble PVP) at an extract/fw ratio of 10:1 essentially as described in Flügge and Weber (1994). Of the extracts 0.6 ml were mixed with 0.5 ml liposome/substrate preparations (see above), frozen in liquid nitrogen and stored at -80°C until further use.

For the assessment of dependencies of PT transport activities from pH-values inside the liposomes, titration curves with K-gluconate, KH_2_PO_4_, and K-PEP were constructed to determine the exact quantity of KOH required to adjust the desired pH-values. Likewise both extraction medium (in the absence of BSA) and liposome buffer were also prepared at the desired pH-values. In this case TRICINE was replaced by a combination of the three buffer substances MES, HEPES, and TRICINE at a molar ratio of 1/3 each. Similarly, for dependencies of TP transport activities from the PEP concentration inside the liposomes, final PEP concentrations in the assays, between 1 and 30 mM, were balanced with K-gluconate to maintain the total concentrations of both compounds of 30 mM.

The frozen extract/liposome mixtures were thawed slowly on ice to initiate the formation of proteoliposomes, which was completed by a second round of sonication with 30 pulses at 30% duty cycle, output control 5. In order to remove the counter-exchange substrate from the external solution, the proteoliposome preparations were subjected to gel filtration on PD-10 columns (Sigma Aldrich) equilibrated with PD-10-medium containing, 100 mM Na-gluconate, 50 mM K-gluconate, and 10 mM Tricine-KOH (pH 7.6). For studying the effect of the external pH of the proteoliposomes on PEP or P_i_ counter-exchange rates, the PD-10-medium was adjusted to the desired pH-value. In case of steep pH-gradients between the internal and external space of the liposome, care was taken that the PD-10 step and the subsequent transport experiments were done within a time frame of less than 3 min.

Transports were initiated by mixing 850 μl proteoliposomes with 50 μl ^32^P-labeled P_i_ solution at pH 7.5 (final P_i_ concentration 0.14 mM) with a specific activity of between 40,000 and 60,000 cpm⋅nmol^-1^ P_i_. For a time-course of P_i_ counter-exchange, transport reactions were terminated after 15, 30, 45, and 60 s by passing 200 μl aliquots of the reaction mixtures through Dowex anion exchange columns (AG1.8, mesh size 100–200; Bio-Rad, Munich, Germany) equilibrated with 0.2 M Na-acetate. For dependencies of transport rates from substrate or inhibitor concentrations, either substrate or inhibitor was contained in the 50 μl labeling solution, to give the desired final concentration after initiating the transport by the addition of the proteoliposomes. Radioactivity taken up by the proteoliposomes was quantified using liquid scintillation counting (Beckman LS7500). Transport rates were linear for at least 1 min. Substrate-specific counter-exchange rates were corrected against transport rates observed for proteoliposomes preloaded with Na-gluconate.

### Detection of the His-Tagged XPT Protein After SDS–PAGE on Immunoblots

The recombinant protein extracted from yeast cells was separated by discontinuous sodium dodecyl sulfate-polyacrylamide gel electrophoresis (SDS–PAGE) according to Laemmli (1970) on 15% separation gels. For immunological detection, proteins were transferred from the SDS-gels to polyvinylidene fluoride (PVDF)-membranes (BioRad, Munich) by electro-blotting according to Jungblut and Seifert (1990) using a ‘semi-dry’ blotting apparatus (Carboglass; Schleicher and Schüll). The membranes were incubated for 2 h in casein containing blocking solution and probed for the detection of the His-tag overnight at 4°C with, mouse anti-penta-His as primary antibody. Following incubation with the secondary antibody, i.e., goat anti mouse conjugated with horse radish peroxidase (Sigma, St. Louis, Missouri), the proteins were detected by chemo-luminescence following the application of SuperSignal West substrate (Thermo Fisher Scientific) in an ImageQuant LAS-4000 Luminescent Analyzer (GE Healthcare).

### Photosynthesis Measurements

Photosynthesis parameters at PSII were determined fluorometrically according to Schreiber et al. (1986) and Genty et al. (1989) with an Imaging PAM (M-series, Maxi version, Walz, Effeltrich, Germany) Induction kinetics of photosynthesis and light saturation curves have been assessed according to the routines implemented in the Imaging PAM software. The plants were dark-adapted for 30 min prior to induction with actinic light for 5 min. Care was taken that the light saturation curves of photosynthesis were measured immediately after induction. The duration at each PFD was 20 s starting with the lowest PFD. The parameters ΦNPQ (quantum efficiency of directed non-photochemical quenching [mainly ‘heat emission’] at PSII) and ΦNO (quantum efficiency of non-directed ‘other’ non-photochemical events) were derived from theoretical considerations discussed in Kramer et al. (2004), simplified by Hendrickson et al. (2004). From the quantum efficiency of PSII electron transport (ΦPSII) non-cyclic electron transport rates (ETR) can be estimated according to Genty et al. (1989). Since neither leaf absorptance nor antenna cross sections of the photosystems and excitation energy distribution between them were determined, ETR is expressed in relative terms.

### Statistical Evaluation of Experimental Data

The data are expressed as mean values ± standard error (SE) of the mean of the indicated number of independent measurements. Significant differences between more than two physiological data sets were analyzed using one-way ANOVA combined with the *post hoc* Tukey-Kramer test, which allows the comparison of unequal sample sizes and identifies values which are significantly different (Ludbrook, 1998). For data plotting and fitting, SigmaPlot10.0 for Windows (Systat Software Inc.) was used. Kinetic constants, *K*_m_ or *K*_i_, were estimated from non-linear curve fits assuming that a hyperbolic response is applicable. The constants were expressed as mean ± SE and derived from between 8 and 10 data points per curve of two independent experiments.

## Results

### The XPT Can Partially Substitute the TPT in Mutants With a Block in Photoassimilate Export From the Chloroplasts

A block in the day- and night-path of carbon export from the chloroplasts would be expected to result in a lethal phenotype. However, the loss of both functions, i.e., TP export by the TPT and starch biosynthesis (and with it starch degradation), resulted only in a severe growth retardation and impaired photosynthesis in the *adg1-1/tpt-2* double mutant (Schmitz et al., 2012 and **Supplementary Figure S1C**), suggesting that additional ways must exist that allow a basic level of carbon export from the chloroplast. The most promising candidate for mediating an alternative carbon export is the XPT. The respective single mutant lacked any visible phenotype different from its wild-type accession (i.e., Ws-2; **Supplementary Figure S1A**). However, double mutants of the TPT and XPT (*tpt-2/xpt-1*) were growth-retarded and impaired in photosynthesis (**Supplementary Figure S1B**). Thus they resembled the *adg1-1/tpt-2* double mutant (**Supplementary Figure S1C**). A detailed study on possible reasons for these unexpected strong growth and photosynthesis phenotypes of the *tpt-2/xpt-1* double mutants is contained in Hilgers et al. (2018). Moreover, *GPT2* expression can be induced in leaves in the presence of high sugar levels (i.e., when plants were grown on Suc; Schmitz et al., 2012; and references therein) and hence GPT2 might also become involved in carbon export from the chloroplasts. The *gpt2-1* single mutant lacks any pronounced growth phenotype when compared to the wild-type (**Supplementary Figure S1B**). Most importantly, For *adg1-1/tpt-2* the additional knockout of GPT2 in the *adg1-1/tpt-2/gpt2-1* triple mutant had also no effect on the partial rescue of the growth and photosynthesis phenotypes, when the plants were grown on sucrose (Schmitz et al., 2012), indicating that GPT2 is not involved in compensational carbon export from the chloroplasts. When grown in soil the triple mutant was indistinguishable from the double mutant (**Supplementary Figure S1C**). For the experiments in this report the knockout of the *GPT2* gene was included again.

The *adg1-1/xpt-1* double mutant was crossed to the *adg1-1/tpt-2/gpt2-1* triple mutant. Both parental lines were viable when grown in soil and exhibited a phenotype similar to the *adg1-1* single mutant or the *adg1-1/tpt-2* double mutant, respectively (**Supplementary Figure S1C**). In the offspring of these crosses a segregating line was isolated that was homozygous for *adg1-1* and *tpt-2*, but heterozygous for the mutations in *XPT* and *GPT2*. Seeds of this line were used for further experiments. Strikingly, we were not able to find any homozygous triple (*adg1-1/tpt-2/xpt-1*) or quadruple mutant (*adg1-1/tpt-2/xpt-1*/*gpt2-1*) amongst the soil-grown plants. In addition, *xpt-1/gpt2-1* double and *adg1-1/gpt2-1/xpt-1* triple mutants were viable when grown in soil (**Supplementary Figures S1B,C**).

**FIGURE 1:**
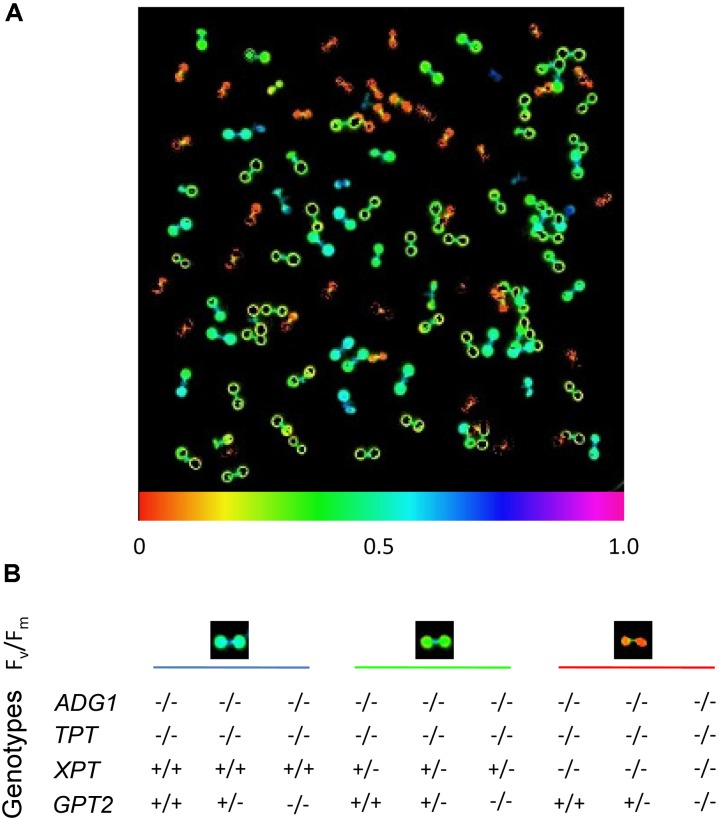
Chl *a* fluorescence phenotype of seedlings from a segregating mutant population defective in *ADG1*, *TPT*, *XPT* and *GPT2*. The plants were germinated and grown on 1/2MS agar plates supplemented with 50 mM sucrose under long-day conditions and a PFD of 150 μmol⋅m^-2^⋅s^-1^ for 8 days. **(A)** Seedlings were dark-adapted for 30 min and the F_v_/F_m_-ratio determined by PAM fluorometry. **(B)** Correlation between the F_v_/F_m_-ratios with the genotype of the mutants (determined on day 28). The F_v_/F_m_-ratio dropped from around 0.5 in *adg1-1/tpt-2* double or *adg1-1/tpt-2/gpt2-1* triple mutants, to around 0.35 when the *xpt-1* mutation was in the heterozygous state and reached 0.06 in homozygous *adg1-1/tpt-2/xpt-1* triple mutants, irrespectively whether *GPT2* was knocked out homo- or heterozygously.

Seeds of the segregating line with the genotype *ADG1*(-/-)/*TPT*(-/-)/*XPT*(+/-)/*GPT2*(+/-) were germi-nated on 1/2 strength MS agar plates supplemented with 50 mM Suc as carbon source. Interestingly, the seedlings could already be distinguished on the basis of their F_v_/F_m_-ratios (**Figure 1**) nine days after sowing. The genotyping, which has been done 28 days after sowing, supported the three different classes of F_v_/F_m_-responses that correlated with the genotypes. The highest F_v_/F_m_-ratio (0.531 ± 0.011; *n* = 5) was observed for plants that were homozygous for the knockout in *ADG1* and *TPT*, but wild type for *XPT*. The F_v_/F_m_-ratio was further significantly diminished to 0.357 ± 0.013 (*n* = 5; *P* < 0.01), when *XPT* was knocked out heterozygously in the *adg1-1/tpt-2* background. Homozygous *adg1-1/tpt-2/xpt-1* triple mutants exhibited the lowest F_v_/F_m_-ratio (0.062 ± 0.012; *n* = 5; *P* < 0.01), suggesting that the capacity of photosystem II is strongly impaired. The additional homo- or heterozygous knockout of *GPT2* had neither an effect on F_v_/F_m_-ratios nor on the growth characteristics of the plants. The homozygous knockout of *ADG1*, *TPT*, and *XPT* resulted in plants that were extremely small even after 28 days of growth on 1/2MS agar plates supplemented with Suc (**Supplementary Figure S2**).

**FIGURE 2:**
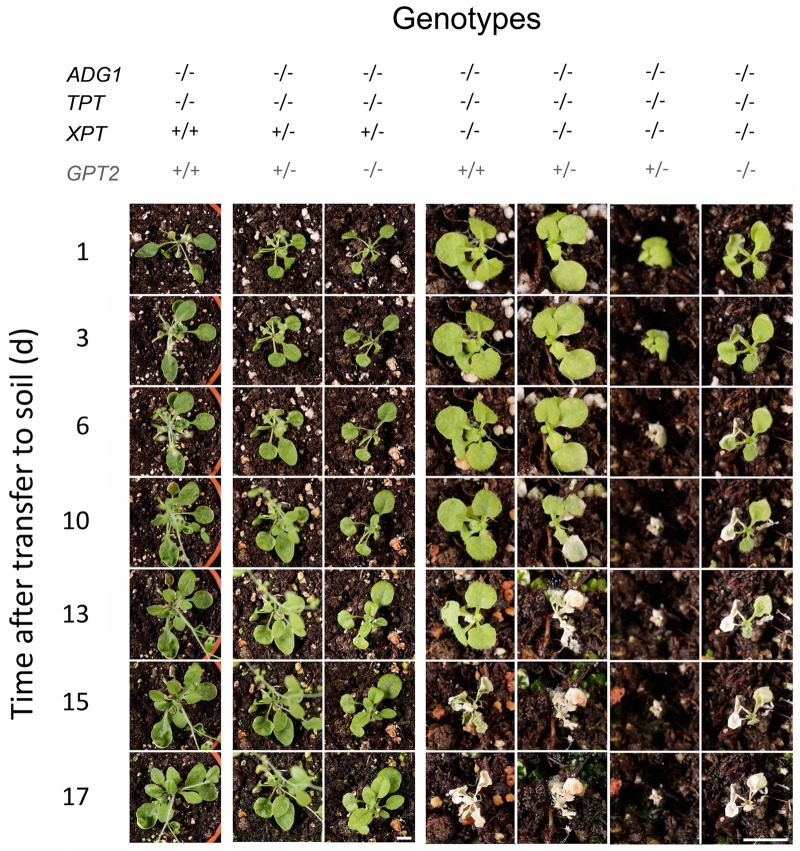
Time course of phenotype developments after transferring members of a segregating mutant population defective in *ADG1*, *TPT*, *XPT* and *GPT2* to soil. Plants were grown for 28 days on 1/2MS agar plates supplemented with 50 mM Suc and then were transferred to soil for the time indicated. The homozygous deficiency of the *XPT* in the *adg1-1/tpt-2* background led to the death of the plants between days 6 and 15 after transfer to soil (left panel), whereas genotypes with a homozygous defect in *ADG1* and *TPT*, but a wild type or a heterozygously affected *XPT* survived on soil (left and central panel). Note the different size of the space bars (representing 1.0 cm) for the right panel and the left/central panel. In all cases plants were grown under long-day conditions at a PFD of 150 μmol⋅m^-2^⋅s^-1^. The zygosity of *GPT2* is shaded in gray because it does not contribute to the overall phenotype.

**FIGURE 3:**
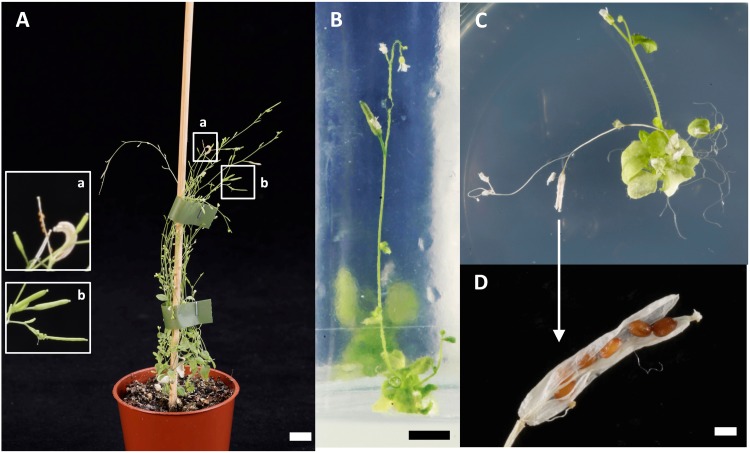
Reproduction of segregating quadruple mutants defective in *ADG1*, *TPT*, *XPT*, *GPT2*. **(A)** A mutant plant carrying a homozygous knockout of *ADG1* and *TPT*, but a heterozygous mutation in the *XPT* is viable and capable of producing siliques and seeds (**a**, ripe siliques with seeds, **b**: green siliques) 32 days after transfer to soil (the bar represents 1 cm). **(B)** A mutant plant homozygous for the mutations in *ADG1*, *TPT*, and *XPT* can only survive on a medium containing 50 mM Suc as carbon source (the space bar represents 0.5 cm). **(C)** At day 115 after sowing the same plant depicted in **(B)** produced some siliques with a small number of seeds. **(D)** Close-up of an opened silique with seeds shown in **(C)** (the space bar represent 1 mm).

After 29 days of growth on agar plates, eight plants homozygous for the mutation in *ADG1*, *TPT*, and *XPT* were transferred to soil together with control plants carrying either a wild-type *XPT* or a heterozygous mutation in the *XPT* (**Figure 2**). All homozygous *adg1-1/tpt-2/xpt-1* triple mutants died within 15 days after transfer to soil. The right panel of **Figure 2** shows four examples of this genotype. In a control experiment, 22 triple mutants were again transferred to 1/2MS agar supplemented with 50 mM Suc, either in jelly jars, Magenta boxes with an air filter, or in open jelly jars placed in Microboxes. All these plants survived for at least another 28 days. Of these plants, 17 developed inflorescences 86 days after germination, but only 7 plants exhibited flowers (**Figure 3B**). Only one plant, grown in an open jelly jar within the Microbox developed siliques with some seeds at day 115 after germination (**Figures 3C,D**). Genotypes with a wild-type like *XPT* or heterozygously mutated *XPT* developed seeds after transfer to soil within 60 days after germination (**Figure 3A**).

These data clearly demonstrate that a combined absence of the TPT and XPT is lethal in a starch-free background, irrespectively of the presence or absence of GPT2, and thus supports the idea that (1) the XPT is capable of partially substituting the TPT as has been proposed previously (e.g., Häusler et al., 2009; Schmitz et al., 2012) and (2) that plants with a complete block in the day and night path of carbon cannot survive.

### Can the XPT Functionally Support the PPTs?

Besides of its main transport substrate, Xu5P, as well as TPs, the XPT can also transport PEP, albeit with a lower V_max_ and a relatively high *K*_m_ as compared to TPs (**Table 1**). Moreover, in contrast to the *TPT*, which is a single copy gene in *A. thaliana*, two *PPT* genes are present in the *A. thaliana* genome, *PPT1* and *PPT2*. *PPT1* is knocked out in several *cue1* mutant alleles (Li et al., 1995). However, so far there are no reports on knockout mutants in the *PPT2* gene available. Here we intend to block PEP transport across the chloroplast envelope simultaneously, i.e., by defects in PPT1, PPT2 and XPT functions. According to **Table 1** both GPTs of *A. thaliana* have also the capacity to transport PEP (with unknown *K*_m_-values). Unlike both *GPT* genes, the *XPT* is ubiquitously expressed and would hence be able to functionally support PPT functions in most, if not all tissues (*efp* browser; Winter et al., 2007). A complete restriction of PEP supply to the stroma should result in an inhibition of the shikimate pathway as it is, for instance, achieved by the application of the herbicide glyphosate, an inhibitor of 5-enolpyruvylshikimat-3-phosphat synthase EPSPS (Steinrücken and Amrhein, 1980).

On the way to block PEP transport across the envelope, we (1) analyzed kinetic properties of the recombinant XPT protein from *A. thaliana* expressed in yeast cells with respect to PEP. (2) Two allelic mutants of *PPT2* (*ppt2-1* and *ppt2-2*) were isolated and established as homozygous lines and (3) triple mutants defective in both PPTs and the XPT functions (*cue1-6/ppt2-1/xpt-1*) were generated by genetic crosses and initially characterized.

### PEP Transport Catalyzed by Recombinant XPT and in Leaf Extracts Is Higher at pH 7.0 and Below

The capacity of PEP transport mediated by the XPT is a critical prerequisite for any further evaluation of its role in supporting both PPTs. In transport studies using the proteoliposome system, the pH of the preloaded counter-exchange substrate is usually adjusted to pH 7.8, which is between the expected neutral pH in the cytosol of the mesophyll and the alkaline pH in the stroma of illuminated chloroplasts (Fischer et al., 1997; Kammerer et al., 1998; Eicks et al., 2002). In earlier studies it could be demonstrated that the TPT is specific for substrates with two rather than three negative charges (Fliege et al., 1978). This charge specificity explains why 3-PGA is a poor export substrate from chloroplasts, because at a pH of about 8.0 in the stroma it exists to 89% in the 3-fold negatively charged form (because of the two negative charges of the phosphate group), whereas TPs are predominantly 2-fold negatively charged (because they do not carry any additional organic acid function). PEP differs significantly from TP and 3-PGA in the p*K*_a_-value for the second proton (i.e., p*K*_a_ = 7.1 for 3-PGA and p*K*_a_ = 6.4 for PEP^5^). Even at pH 7.0, PEP exists to 80% in the 3-fold negatively charged form compared to 3-PGA with only 44%.

**FIGURE 4:**
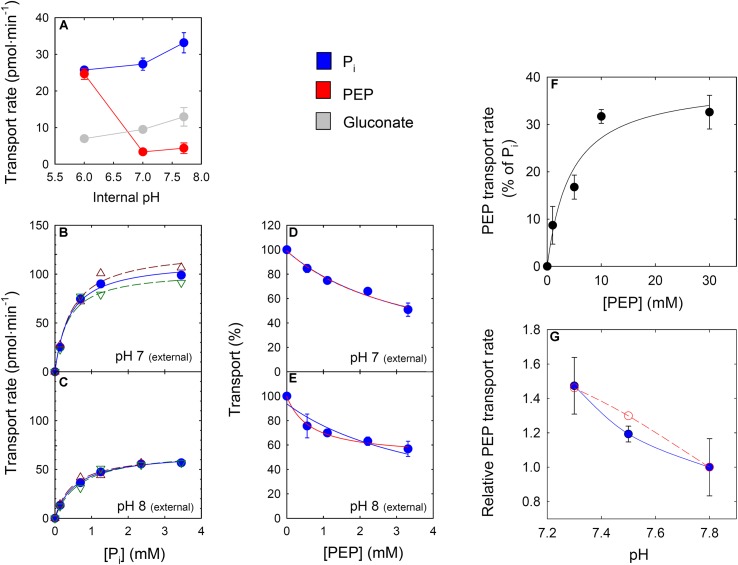
Kinetic properties of the heterologously expressed XPT protein or of leaf extracts in the proteoliposome system. In **(A)** the internal space of the proteoliposomes was preloaded either with PEP (red circles), P_i_ (blue circles) or gluconate (light grey circles) (30 mM each) adjusted to pH values of 7.8, 7.0 and 6.0. The transport kinetic was initiated by adding 0.14 mM ^32^P labeled P_i_ as a counter-exchange substrate at pH 7.5. In **(B,C)** substrate saturation kinetics with radiolabeled P_i_ at either pH 7.0 or pH 8.0 outside of the proteoliposomes (inside pH 7.8) are shown. The red or green open triangles represent individual measurement, whereas the closed circles indicate the mean values. In **(D,E)** inhibition kinetics of P_i_ import (0.14 mM) by increasing concentrations of PEP are shown. Curves were fitted to the experimental data (mean of two experiments) under the assumption that the hyperbolic decay curve approaches a transport rate of zero at infinite PEP concentrations (blue line) or the decay curve intercepts the transport axis in the positive range (red line). Both curve fits resulted in an identical **(D)** or a substantial different curvature **(E)**. In **(F)** proteoliposomes containing membrane proteins from Col-0 leaf extracts were preloaded with increasing PEP concentrations balanced with gluconate to maintain a constant osmolarity. P_i_ counter-exchange was initiated by the addition of ^32^P-labeled P_i_ (0.14 mM). From the hyperbolic curve fit a *K*_m_-value for PEP of 4.34 ± 2.6 mM was determined. In **(G)** a dependency of P_i_/PEP counter-exchange from the internal pH inside the proteoliposomes at a total PEP concentration of 3 mM is shown. The blue symbols and line indicate the experimental data, whereas the red circles and line were predicted for counter-exchange rates, assuming that two-fold negatively charged PEP^2-^ represents the transported PEP species.

According to Eicks et al. (2002) the maximum transport activity of the XPT for PEP was about 10% of the P_i_ transport with a relatively poor *K*_m_ for PEP compared to the PPT from cauliflower buds (see **Table 1**). In order to test whether the XPT is a suitable candidate for PEP transport, the recombinant XPT protein was expressed in yeast (**Supplementary Figure S3A**) and the isolated yeast membranes carrying the XPT protein were reconstituted into proteoliposomes. The homologous ^32^P_i_/^31^P_i_ counter-exchange rate was linearly correlated with the amount of yeast membranes (**Supplementary Figure S3B**). As it was intended to analyze the transport characteristics of the XPT in a pH-dependent manner, the internal space of the proteoliposomes was adjusted to three different pH-values. **Figure 4A** shows that PEP, compared to P_i_, is only poorly transported at pH-values between 7 and 7.7. In contrast, at pH 6.0, PEP transport increased dramatically, and the absolute rates of PEP- and P_i_-transport were very similar. In contrast to PEP, P_i_-transport showed only a weak response toward changes in the internal pH-values. Hence, depending upon the pH of the surrounding medium, PEP can be transported more efficiently by the XPT than expected from previous studies.

A further focus of our study was the determination of kinetic constants of the XPT at two different pH-values of the external medium of the proteoliposomes. Substrate saturation kinetics of the recombinant XPT with P_i_ (**Figures 4B,C**) and inhibition kinetics of the homologous ^32^P_i_/^31^P_i_ exchange by increasing PEP concentrations (**Figures 4D,E**) were constructed at pH 7.0 and 8.0. Interestingly, the *K*_m_-values for P_i_ lacked any pronounced dependency on the pH (i.e., 0.40 ± 0.07 mM at pH 7.0; or 0.57 ± 0.05 mM at pH 8.0) albeit the V_max_ decreased from 114 pmol⋅min^-1^ at pH 7.0 to 67 pmol⋅min^-1^ at pH 8.0. Initial inhibition studies of homologous ^32^P_i_/^31^P_i_ transport by applying increasing PEP concentrations to the external medium revealed an interesting aspect. Whilst at pH 7.0 the transport data fitted well to a decaying hyperbola approximating zero at infinite PEP concentration (**Figure 4D**), at pH 8.0 this was not the case (**Figure 4E**). The latter data set fitted much better to a decaying hyperbola with an offset >0 (**Figure 4E**; red line) rather than zero (**Figure 4E**; blue line). Interestingly, with both fits identical curves were obtained for pH 7.0 (**Figure 4D**), but not at pH 8.0. At pH 7.0, PEP binds with an apparent *K*_i(app)_-value of 3.76 ± 0.39 mM, whereas at pH 8.0 two *K*_i(app)_-values could be obtained depending on the curve fit. The decaying hyperbola without offset provided a K_i(app)_ of 3.71 ± 0.7 mM PEP and the decaying hyperbola with offset >0 delivered a K_i(app)_ of 0.68 ± 0.07 mM PEP, suggesting different modes of substrate/inhibitor binding. However, more detailed studies are required to unravel possible differences in substrate binding mechanisms with changing pH-values (Segel, 1993).

Based on these findings both the concentration dependency and the pH-response of PEP transport were further analyzed with extracts of wild-type leaves. Proteoliposomes containing reconstituted membrane proteins from Col-0 leaves were preloaded with PEP concentrations of between 1 and 30 mM (balanced with gluconate) at an internal pH of 8.0 and the counter-exchange rate with radio-labeled P_i_ was measured (**Figure 4F**). The saturation kinetics exhibited a hyperbolic response between transport rates and substrate concentrations with an apparent *K*_m_-value for PEP export of 4.34 ± 2.14 mM. If it is considered that PEP^2-^ might be the transported substrate species, the *K*_m_-value for PEP^2-^ would be 0.106 ± 0.053 mM at pH 8.0. Moreover, as is shown in **Figure 4G**, there was a substantial increase of PEP/P_i_ counter-exchange with decreasing pH-values, suggesting that the pH-dependency seems to be a general feature of PEP transport catalyzed by the PPTs and the XPT. Using the *K*_m_-value for PEP^2-^ deriving from the substrate saturation curve in **Figure 4F** and the PEP^2-^ concentrations in equilibrium at the three different pH-values depicted in **Figure 4G**, it is possible to construct the dependency of PEP/P_i_ counter-exchange rates from the PEP^2-^ concentration, represented by the red open symbols and the red dashed line (**Figure 4G**). It is hence conceivable that the pH-dependency of PEP/P_i_ counter-exchange is governed by the availability of PEP^2-^ as transport substrate.

### Mutants Deficient in PPT2 Are Slightly Retarded in Growth Compared to Wild-Type Plants

Until now there are no reports on *A. thaliana* mutants deficient in PPT2. Two approaches were deployed to identify different alleles of PPT2 knockout mutants, (1) the GABI-TILLING program and (2) the search for T-DNA insertion mutants.

**FIGURE 5:**
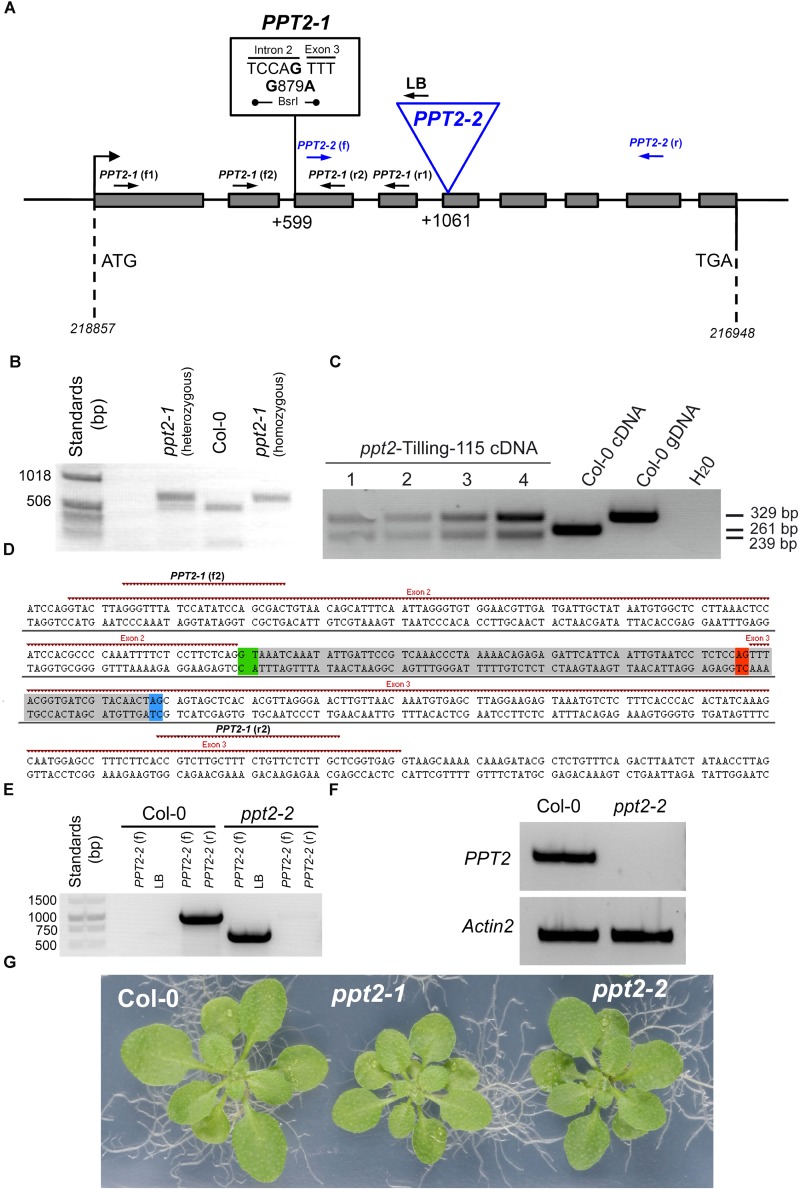
Molecular characterization of mutants deficient in PPT2. **(A)** *PPT2* gene structure with individual mutation either deriving from the TILLING approach (i.e., EMS mutagenized lines), *ppt2-1*, or from a T-DNA insertion, *ppt2-2*, as well as the binding sites of primer pairs for the identification of the mutations. For screening of *ppt2-1* **(B)**, which is characterized by a mutated splice motive in front of exon 3, advantage was taken that the base pair exchange in the mutant also destroyed a binding site for the restriction enzyme Bsr*I*. Genomic DNA, amplified by the gene specific primers *ppt2-1* (f1) and *ppt2-1* (r1) delivered either two bands, in the heterozygous state, or a larger and smaller band for the mutant and wild type, respectively, after digestion with Bsr*I*. **(C)** *PPT2* transcripts (cDNA) in the *ppt2-1* mutant (= *ppt2*-Tilling-115) revealed two bands as compared to only one band in Col-0 *PPT2* cDNA or gDNA. This double band is based in an alternative splice site upstream of the mutated splice site when the primer pairs *ppt2-1* (f2) and *ppt2-1* (r2) were used for amplification and the products were digested with Bsr*I* (the numbers 1 to 4 denote increasing DNA amounts). **(D)** The genomic DNA region of the *PPT2* gene, which was amplified by PCR using the primer pairs *ppt2-1* (f2) and *ppt2-1* (r2), reveals the two splice sites in front (green) and at the end (red) of the second intron. The latter is mutated in the *ppt2-1* mutant and gives rise to the use of an alternative splice site (blue) instead of the mutated site, leading to the partial excision of the gray-shaded DNA region and the occurrence of the characteristic double bands of the *ppt2-1* mutant. In **(E)** the identification of the homozygous *ppt2-2* T-DNA insertion mutant with genomic DNA isolated from Col-0 wild-type and *ppt2-2* is shown. The primer pairs used are indicated in the Figure. **(F)** The homozygous *ppt2-2* mutant lacks any transcripts (cDNA) of *PPT2* as compared to *Actin2.* For the amplification the *ppt2-2* (f) and (r) primers were used. **(G)** Rosette phenotypes of wild-type and *ppt2* mutant plants grown for three weeks on 1/2MS agar plates at a PFD of 150 μmol^-2^⋅s^-1^ in the long-day.

For the TILLLING approach, among 14 candidate lines with mutations in the desired region there was only one promising candidate for a knockout in the *PPT2* gene. The line 115 carried a base pair exchange at position 879, (*A* against *G*; i.e., at position 599 upstream of the translational start codon), directly in front of the third exon (**Figure 5A**). By this mutation a splice site was eliminated resulting in an incorrect splicing of the second and third exon, i.e., the second intron remained still part of the mRNA (or cDNA). Apart from the mutation in the splice site, the base exchange at position 879 also destroyed a recognition site of the restriction enzyme Bsr*I*, which offered a way to identify homozygous mutants by a restriction digest (**Figure 5B**). Moreover, by sequencing the different PCR products shown in **Figure 5C**, it became evident that, due to incorrect splicing at the mutated splice site, a secondary splice site with the same sequence 22 bp downstream of position 897 was used in the mutant (**Figure 5D**). However in both cases, i.e., the mutated and the alternative splice sites, the predicted amino acid sequences of the resulting proteins would contain translational stop codons after 166 or 142 amino acids, respectively (**Supplementary Figure S4**). A translational stop in this region would result in an impaired protein between the second and third transmembrane domain. The *ppt2-1* mutant was backcrossed to the wild type three times to shed additional point mutants resulting from the EMS treatment.

For the second approach, a mutant was isolated carrying the T-DNA insertion at position 1061 bp downstream of the start codon, right at the beginning of the 5^th^ exon (**Figures 5A,E**). The *ppt2-2* mutant lacked any transcript, when gene specific primers were used to amplify the region before and behind the insertion site (**Figure 5F**).

The rosettes of both mutant alleles appeared slightly smaller compared to the wild type (**Figure 5G**). Growth rates based both on rosette areas and leaf fresh weights revealed small, but significant growth retardations for both mutant alleles compared to wild-type plants at day 21 after sowing (**Supplementary Figure S5**) and hence support **Figure 5G**. A statistical analysis is contained in **Supplementary Table S2A**. The Chl *a* fluorescence characteristics exhibited only minor differences between both *ppt2* alleles and the wild type (**Table 2**). Induction and light curves of relative ETR as well as efficiencies of PSII (ΦPSII) and non-photochemical components (ΦNPQ and ΦNO) were also very similar between the mutant and the wild-type plants (**Supplementary Figure S6**). However, some small differences were significant due to low errors and high sample sizes (**Supplementary Table S2B**).

**Table 2:** Chl fluorescence parameters of wild-type and mutant plants defective in PPT2.

Plant lines	*F*_v_/*F*_m_	ΦPSII_(164)_	Relative ETR_(800)_
Col-0	0.760 ± 0.003	0.509 ± 0.006	80.9 ± 2.3
*ppt2-1*	0.760 ± 0.003	0.497 ± 0.006	74.3 ± 2.0
*ppt2-2*	0.763 ± 0.003	0.535 ± 0.005	84.5 ± 2.6


*The plants were grown for three weeks in soil at a PFD of 150 μmol⋅m^-*2*^⋅s^-*1*^ in the long-day. The PFD during the induction of photosynthesis and the determination of ϕPSII was 164 μmol⋅m^-*2*^⋅s^-*1*^. The relative ETR_*(800)*_ determined at the highest PFD applied (i.e., 800 μmol⋅m^-*2*^⋅s^-*1*^) was obtained from light curves (**Supplementary Figure S6**). The data represent the mean ± SE of *n* = 15 replicates.*

### The Additional Knockout of PPT2 and the XPT Had No Effect on the Vegetative Phenotype of the *cue1-6* Mutant

A deficiency in PPT1 results in a strong growth retardation and a reticulate leaf phenotype of the *cue1* mutant, which is still not completely understood. Here we asked the question as to which extent additional PEP transport activity mediated by PPT2 and/or the XPT guarantees survival of the *cue1* mutant.

**FIGURE 6:**
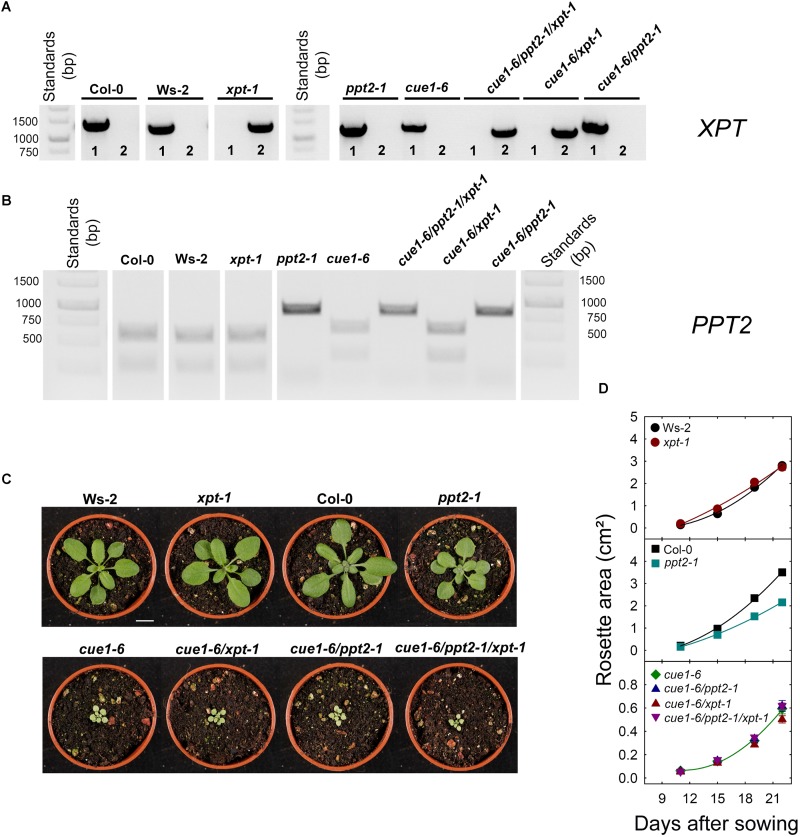
Molecular and phenotypic analyses of wild-type and mutant plants impaired in the *PPT* genes and/or the *XPT*. In **(A,B)** the identities of the mutation in *XPT* and *PPT2* were confirmed with genomic DNA. The numbers in **(A)** denote PCR with *XPT-1* (f) and (r) primers to identify wild-type *XPT* (1) or PCR with LP*_XPT-1_* (f) and *XPT-1* (r) primers to identify to insertion in *xpt-1* (2) (see **Supplementary Table S1**). In **(C)** the phenotypes of plants grown in soil for 20 days after germination in a temperature controlled growth chamber at a PFD of 150 μmol^-2^⋅s^-1^ are shown. The bar represents 1 cm. **(D)** Growth rates of leaf rosettes are shown for Ws-2 (black circles), *xpt-1* (dark red circles), Col-0 (black squares), *ppt2-1* (dark cyan squares), *cue1-6* (dark green diamonds), *cue1-6/ppt2-1* (dark blue triangles), *cue1-6/xpt-1* (dark red triangles) and *cue1-6/ppt2-1/xpt-1* (dark purple triangles). The plants were grown on 1/2MS agar plates at a PFD of 150 μmol^-2^⋅s^-1^ in the long-day. The data represent the mean ± SE of *n* = 15 to 16 replicates. A statistical analysis of growth parameters is contained in **Supplementary Table S3A**.

**Figure 6** shows the characterization of crosses between *cue1-6*, *ppt2-1*, and *xpt-1* compared to the wild type. Based on genomic DNA the mutation in *XPT* (**Figure 6A**) or *PPT2* (**Figure 6B**) could be verified. Surprisingly, the combined knockouts of *PPT2* and the *XPT* in the *cue1-6* background (i.e., *cue1-6/ppt2-1*, *cue1-6/xpt-1*, and *cue1-6/ppt2-1/xpt-1*) had no obvious additional effect on the *cue1-6* phenotype (**Figure 6C**). The first visual impression was quantified as time-dependent increase in the leaf area between days 11 and 22 after sowing of around 16 plants per line. As is shown in **Figure 6D** there were no significant differences in growth between *cue1-6* and the respective *cue1-6/ppt2-1* and *cue1-6/xpt-1* double mutants or the *cue1-6/ppt2-1/xpt-1* triple mutant. In general, growth rates of mutant plants in the *cue1-6* background were much smaller as compared to the respective wild-type or single mutant plants with the exception of *ppt2-1*, which also showed a significantly smaller increment in growth. The above data indicate that a single knockout of the *PPT2* gene seems to be able to slightly diminish growth, but this effect is not additive with the growth retardation in *cue1-6*. Hence neither PPT2 nor XPT are likely to significantly support survival of *cue1-6*.

### Generative Development Reflects Constraints in Vegetative Growth of PPT2 and XPT Mutants in the *cue1-6* Background

The generative development of wild-type and mutant plants impaired in both PPTs and/or the XPT is summarized in **Table 3**. A general characteristic of *cue1-6* and further mutants in this background was a diminished shoot height (compare **Supplementary Figure S7**). The number of siliques, as one of the major characteristics of generative development, was diminished by at least 50% in all lines with a *cue1-6* background. However, a detailed analysis of more subtle differences in generative growth parameters between mutants in the *cue1-6* background was hampered by the fact that both wild-type accessions already showed large variations. A statistical analysis of the data is contained in **Supplementary Table S3A**.

**Table 3:** Generative growth of wild-type plants and mutants impaired in the PPT and XPT.

	Shoot height(cm)	Silique number per plant
Ws-2	38.40 ± 1.00	260.6 ± 13.7
*xpt-1*	42.13 ± 0.85	320.0 ± 10.0^∗^
Col-0	37.79 ± 1.03	187.6 ± 14.3
*cue1-6*	18.44 ± 1.05^∗^	43.8 ± 5.4^∗^
*ppt2-1*	35.46 ± 1.52	167.1 ± 12.5
*cue1-6/ppt2-1*	20.21 ± 1.45^∗^	89.8 ± 18.7^∗^
*cue1-6/xpt-1*	25.33 ± 0.84^∗^	74.4 ± 9.5^∗^
*cue1-6/ppt2-1/xpt-1*	16.73 ± 2.22^∗^	41.5 ± 10.2^∗^


*Plants were grown in soil at a PFD of 150 μmol⋅m^-*2*^⋅s^-*1*^ in the long-day. Shoot height and silique number of the plants were determined 49 days after sowing (**Supplementary Figure S7**). The data represent the mean ± SE of *n* = 8 to 9 replicates. Significant differences with respect to the individual wild-type plants are indicated by stars. A statistical analysis of the data is contained in **Supplementary Table S3A**.*

### Photosynthesis Was Slightly More Hampered in *cue1-6/ppt2-1/xpt-1* Triple Mutants Compared to *cue1-6*

Photosynthesis parameters in the individual lines were determined by PAM fluorometry (**Table 4A**). Apart from expected differences in photosynthesis between *cue1-6* and wild-type plants as well as the *xpt-1* and *ppt2-1* single mutants, there were also some small, but significant drops in the F_v_/F_m_-ratio and ΦPSII_(164)_ between the *cue1-6/ppt2-1/xpt-1* triple mutant and the *cue1-6* single mutants. Hence a combined knockout of both PPTs and the XPT had a subtle impact on photosynthesis in the *cue1-6* background.

**Table 4:** **(A)** Chl fluorescence parameters and **(B)** pigment and protein contents as well as specific leaf fresh weights of wild-type and mutant plants defective in the XPT and PPT1 or PPT2.

A
**Plant lines**	***F*_v_/*F*_m_-ratio**		**ΦPSII_(164)_**		**Relative ETR_(800)_**	

Ws-2	0.760 ± 0.002		0.492 ± 0.007		80.8 ± 2.3	
*xpt-1*	0.769 ± 0.002		0.498 ± 0.003		85.6 ± 3.9	
Col-0	0.753 ± 0.002		0.493 ± 0.007		81.4 ± 3.1	
*cue1-6*	0.704 ± 0.004^∗^		0.439 ± 0.005^∗^		58.2 ± 1.9^∗^	
*ppt2-1*	0.757 ± 0.003		0.510 ± 0.003		83.3 ± 1.9	
*cue1-6/xpt-1*	0.708 ± 0.009		0.420 ± 0.016		57.1 ± 3.9^∗^	
*cue1-6/ppt2-1*	0.699 ± 0.006^∗^		0.457 ± 0.009^∗^		58.5 ± 1.7^∗^	
*cue1-6/ppt2-1/xpt-1*	0.655 ± 0.009^∗^		0.374 ± 0.017^∗^		46.4 ± 2.3^∗^	

**B**

**Plant lines**	**Chl content**	**Carotenoid content**	**Chl *a/b*-ratio**	**Chl/Car-ratio**	**Protein content**	**Specific fw**
				
	**(mg⋅m^-2^)**				**(g⋅m^-2^)**	

Ws-2	192.29 ± 10.53	19.97 ± 2.40	3.18 ± 0.03	10.16 ± 1.25	3.51 ± 0.08	206.18 ± 4.35
*xpt-1*	194.94 ± 4.62	20.14 ± 1.66	3.17 ± 0.08	9.88 ± 0.64	3.66 ± 0.28	217.67 ± 5.98
Col-0	200.60 ± 4.69	22.23 ± 0.62	3.20 ± 0.07	9.04 ± 0.25	3.39 ± 0.26	242.57 ± 14.19
*cue1-6*	115.13 ± 3.07^∗^	21.92 ± 0.93	3.23 ± 0.27	5.28 ± 0.24^∗^	3.11 ± 0.51	166.80 ± 10.91^∗^
*ppt2-1*	203.51 ± 7.74	19.38 ± 1.45	3.10 ± 0.01	10.70 ± 0.75	3.30 ± 0.09	230.56 ± 4.92
*cue1-6/ppt2-1*	127.48 ± 9.36^∗^	22.33 ± 1.20	2.72 ± 0.07	5.71 ± 0.33^∗^	3.07 ± 0.28	178.73 ± 12.70^∗^
*cue1-6/xpt-1*	135.50 ± 8.47^∗^	23.82 ± 0.61	2.79 ± 0.13	5.67 ± 0.23^∗^	3.23 ± 0.50	171.91 ± 12.21^∗^
*cue1-6/ppt2-1/xpt-1*	109.96 ± 7.26^∗^	21.58 ± 1.37	2.83 ± 0.19	5.10 ± 0.13^∗^	3.06 ± 0.27	164.49 ± 13.35^∗^


*The plants were grown for three weeks in soil at a PFD of 150 μmol⋅m^-*2*^⋅s^-*1*^ in the long-day. In **(A)** the PFD during the induction of photosynthesis and the determination of ϕPSII was 164 μmol⋅m^-*2*^⋅s^-*1*^. The relative ETR_*(800)*_ determined at the highest PFD applied (i.e., 800 μmol⋅m^-*2*^⋅s^-*1*^) was obtained from light curves (**Supplementary Figure S8**). The data represent the mean ± SE of *n* = 9 replicates **(A)**, *n* = 4 to 10 replicates **(B)**. Significant differences with respect to the individual wild-type plants are indicated by stars. A statistical analysis of the data is contained in **Supplementary Tables S3B,C**.*

Moreover, the induction kinetics and light curves of photosynthesis parameters, including the efficiencies of directed and undirected non-photochemical processes, i.e., ΦNPQ and ΦNO, are shown in **Supplementary Figure S8**. Surprisingly, the diminished ΦPSII in the *cue1-6* background, in particular at PFDs above 400 μmol⋅m^-2^⋅s^-1^, was accompanied by an increase in ΦNO rather than ΦNPQ, suggesting that directed heat emission from the antennae of PSII seems not to be altered as a consequence of a lack in both PPTs and the XPT. The increase in ΦNO was more marked in the *cue1-6/ppt2-1/xpt-1* triple mutant (**Supplementary Table S2B**).

Furthermore, we addressed the question whether changes in ETR are also reflected in different contents and compositions of pigments related to photosynthesis. As might be expected from the reticulate leaf phenotype, total Chl contents dropped by around 38% from an average of 197 mg⋅m^-2^ in the wild type and single mutants to about 122 mg⋅m^-2^ in the *cue1-6* background, leaving the Chl *a/b*-ratio unchanged (**Table 4B**). This drop in Chl reflects quite well the diminished relative ETR of 30%. However, neither carotenoid contents nor Chl *a/b*-ratios or leaf protein levels showed any significant differences between the individual lines. The specific leaf fresh weight determined as mass-to-area-ratio followed basically the decline in Chl contents. Both diminished Chl and specific fresh weights are governed by larger intercellular air spaces in the mesophyll of *cue1-6* (Kinsman and Pyke, 1998).

### Transport Measurements Revealed Residual PEP/P_i_ Counter-Exchange Capacities in *cue1-6/ppt2-1/xpt-1* Triple Mutants

**FIGURE 7:**
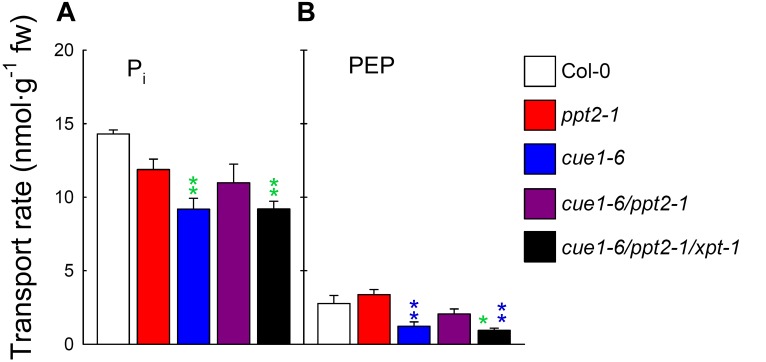
Transport rates for P_i_ and PEP determined with reconstituted membrane proteins from wild-type and mutant plants in the proteoliposome system. In **(A,B)** rates of P_i_ and PEP counter-exchange with 0.14 mM radiolabeled ^32^P_i_ (pH 7.5) are shown for Col-0 wild-type (white bars), the *ppt2-1* (red bars) and *cue1-6* (blue bars) single mutants as well as the c*ue1-6/ppt2-1* double mutant (dark purple bars), and the *cue1-6/ppt2-1/xpt-1* triple mutant (black bars). The rates of the negative control, gluconate (indicating P_i_ uniport), were subtracted from the specific counter-exchange rates with P_i_ or PEP. The data represent the mean ± SE of *n* = 4 replicates. Significant differences with respect to the wild-type or the *ppt2-1* single mutant are indicated by green or blue stars, respectively. *P* < 0.05 or *P* < 0.01 is indicated by single or double stars (of the same color), respectively. A statistical evaluation of the data is contained in **Supplementary Table S4**.

The determination of PEP/P_i_ counter-exchange rates with reconstituted membrane proteins isolated from leaf extracts of wild-type and mutant plants in the proteoliposome system might provide the ultimate answer to the question, whether or not PEP transport across the envelope membrane of plastids is blocked when both PPTs and the XPT are missing. However, as **Figure 7** reveals, this block in PEP transport was not observed. After subtracting the unspecific P_i_ uniport in the presence of gluconate, there was still around 34% PEP/P_i_ counter-exchange detectable in the triple mutant compared to the wild type (**Figure 7B**). Small changes of PEP transport observed between the *ppt2-1* single mutant and Col-0 and between *cue1-6/ppt2-1* and *cue1-6* (**Figure 7B**) were statistically not significant (**Supplementary Table S4**). The same applies for changes in P_i_ transport (**Figure 7A**).

The transport data with single, double, and triple mutants underline the phenotypic appearance of the same lines and suggest that neither the absence of PPT2 nor XPT have a strong effect on PEP deficiency in plastids. From the above experimental approach it appears likely that activities of other PTs are responsible for PEP transport across the envelope.

## Discussion

Here we have focused on the physiological role of TP and PEP transport mediated by the XPT, which are most likely only side activities of the major path, i.e., the retrieval of pentose phosphates from the extraplastidial space (Hilgers et al., 2018). The data in this report demonstrate clearly that (1) the XPT can support or even partially substitute the TPT due to its TP transport capacity. This notion was reinforced by the observation that a complete block of all known ways to export photoassimilates from the chloroplasts, including the XPT, resulted in a lethal phenotype. (2) Most surprisingly, neither the XPT or PPT2 alone, nor in combination, could obviously deteriorate the dramatic growth retardation of the *cue1-6* mutant, indicating that other processes, including transport, support the residual viability of *cue1-6*, in particular in the *cue1-6/ppt2-1/xpt-1* triple mutant. (3) As an additional result of our study, we could show that PEP transport mediated by the recombinant XPT of *A. thaliana* and by extracts from Col-0 leaves could be improved by lower pH-values, suggesting that PEP^2-^ is the transported charge-species. As discussed in more detail below, the data presented here will have wide-reaching implication for our understanding of the functional roles of PTs in various metabolic pathways as well as tissue-type specific developmental processes.

### The XPT Can Support the TPT in the ‘day Path of Carbon’

The beneficial role of the XPT for survival of mutant plants impaired in the day- and night-path of photoassimilate export from chloroplasts could be confirmed in this study. As already expected from theoretical considerations (Häusler et al., 2009; Schmitz et al., 2012, 2014), the triple knockout mutant, *adg1-1/tpt-2/xpt-1*, was not viable anymore. The deficiency in the known ways of carbon export from the chloroplasts resulted in the death of the plants within six to 15 days when grown in soil. Moreover, GPT2, which can be induced by feeding of sugars, was not responsible for the survival of the triple mutant grown on 1/2MS agar plates supplemented with 50 mM Suc. The absence of GPT2 in the *adg1-1/tpt-2/xpt-1* background had no additional negative effect on the already harsh growth phenotype of the triple mutants even in the presence of an external carbon source (compare **Supplementary Figure S2B**). This observation resembled earlier ones made with the *adg1-1/tpt-2/gpt2-1* triple mutant (Schmitz et al., 2012), where the absence or presence of GPT2 had no influence on the sugar-dependent rescue of the *adg1-1/tpt-2* double mutant (Heinrichs et al., 2012; Schmitz et al., 2012). In contrast, here even the heterozygous defect of the *XPT* in the *adg1-1/tpt-2* background resulted in a significant drop in the F_v_/F_m_-ratio by about 33% in seedlings grown on sucrose-containing medium, suggesting a dosage effect of XPT expression on photosynthesis parameters. Still, it remains to be shown whether or not ectopic overexpressions of the XPT or of GPT2 would be capable of completely rescuing the *adg1-1/tpt-2* phenotype.

### Knockouts of PPT2 and the XPT in the *cue1-6* Background Are Viable

The most striking and unexpected outcome of this study were the only minor additional constraints when either or both PPT2 and XPT were knocked out in the *cue1-6* background. Here a lethal phenotype would have been expected, like crop plants show that were treated with the shikimate pathway herbicide glyphosate. However, apart from a slightly restricted relative ETR and an increase in ΦNO, as non-photochemical quench parameter, vegetative growth was not stronger affected in the *cue1-6/ppt2-1/xpt-1* triple mutant as compared to *cue1-6* or the *cue1-6/ppt2-1* and *cue1-6/xpt-1* double mutants. Moreover, comparisons of generative development between the lines was complicated by the fact that both wild-type accessions already showed larger differences in e.g., silique numbers per plants. Using different alleles of the *xpt* mutant for genetic crosses and/or artificial microRNA:*XPT* approaches in single or double mutant backgrounds Hilgers et al. (2018) might eliminate such unwanted side effects in future studies, and more subtle differences in various aspects of growth will probably show up. However, more important compared to subtle effects on growth between the lines was the observation that the triple mutants still exhibited substantial PEP transport capacities, which were similar to those of the *cue1-6* single mutant. Again potential differences between transport rates of individual wild-type accessions (see Kunz et al., 2010) have not been assessed in this experiment.

The second unexpected outcome was the only moderate growth phenotype of both knockout alleles of PPT2 (i.e., *ppt2-1* and *ppt2-2*) leaving photosynthesis, leaf constituents, or transport unaffected.

PEP provision to plastids not only relies on import, but also on pathways capable of generating PEP inside the plastids (Flügge et al., 2011). The most prominent pathway is glycolysis involving a plastidial enolase (ENO1). In an earlier report we could demonstrate that *ENO1* is absent from mature leaves of *A. thaliana*, but can be present in generative and meristematic tissues (Prabhakar et al., 2009; and references therein). Indeed the absence of PEP provision to plastids in certain developmental stages could lead to lethality of gametophytes or embryos, as has been demonstrated for *cue1-6/eno1* double mutants (Prabhakar et al., 2010). These data support the importance of plastidial PEP for plant development and metabolism, but they also showed that other transporters, like PPT2 could not compensate for a deficiency in PPT1, particularly not in *cue1-6/eno1* double mutants. Moreover, additional ways to produce PEP from pyruvate, for instance *via* a plastidial localized pyruvate phosphate dikinase (PPDK) could also be ruled out in the **cue1-6/eno1** double mutant. This was surprising because the *cue1* mutant phenotype could be rescued both by overexpression of a heterologous PPT from cauliflower buds and a C3-type PPDK from *Flaveria pringlei* targeted to the plastids (Voll et al., 2003), indicating the crucial role of plastidial PEP in the development of the *cue1* phenotype. Moreover, *cue1* could also be complemented by constitutive overexpression of the homologous *A. thaliana* PPT1, but only partially by PPT2 (Knappe et al., 2003). The latter experiment casts some doubt on functional similarities between PPT2 and PPT1, i.e., both translocators differ, for instance, in their capacities to transport PEP or 2-PGA (**Table 1**). Moreover, for tissues that contain a complete plastidial glycolysis, the PPT might act as overflow valve for cytosolic or plastidial PEP (Staehr et al., 2014). Whilst in chloroplasts the PPT might function as a net PEP importer based on concentration gradients across the inner envelope membrane, in root plastids, PEP might be exported, again based on concentration gradients and the demand for glycolytic intermediates in both compartments.

Still, internal ways to produce PEP in plastids cannot explain the substantial PEP transport rate detected in the**cue1-6/ppt2-1/xpt-1** triple mutants. It is hence tempting to speculate that other PTs apart from both PPTs and the XPT might also be involved in PEP transport. The most promising candidates are both GPTs, which exhibited maximum PEP transport capacities of up to 40% of the P_i_ transport (**Table 1**). However, *K*_m_-values for PEP have not been determined for the GPTs of *A. thaliana*. For the GPT from *Pisum sativum* roots, the *K*_m_-value for PEP was in the same order of magnitude as for the XPT from *A. thaliana*. In order to support or even functionally substitute each other, the respective PTs have to be present in the same tissue. For both GPTs of *A. thaliana*, *GPT2* is usually not expressed in leaves and *GPT1* expression is restricted to the mesophyll adjacent to the vasculature (bundle sheath) and the stomatal guard cells. The latter has been shown in *A. thaliana* plants expressing a *GPT1* promoter::GUS construct Niewiadomski et al. (2005). Moreover, the functionality of GPT1 has been indirectly demonstrated by the fact that double mutants deficient in GPT2 (to avoid Glc6P-transport in addition to that catalyzed by GPT1) and plastidial phosphoglucose isomerase (PGIp) produced starch granules both in the bundle sheath and in guard cells, whereas the mesophyll was starch-free (Kunz et al., 2010). Starch biosynthesis in leaves hence relies on the import of Glc6P from the cytosol in the absence of PGIp. In this study a functional *GPT1* in the triple mutant might be sufficient for the detected PEP transport rate shown in **Figure 7**. Furthermore, as all *cue1* alleles are characterized by a reticulate leaf phenotype with large intercellular air spaces in the mesophyll, but intact bundle sheath (Kinsman and Pyke, 1998), the functional GPT1 might even be overrepresented in the *cue1-6* background as compared to the wild-type and other mutant plants.

### PEP Transport Is Improved by pH-Values Close to Neutral and Below

Here the kinetic studies with the recombinant XPT confirmed data by Eicks et al. (2002) that, in principle, the XPT is capable of transporting PEP. Moreover, as shown in the present report, the PEP transport rate could be substantially increased with a decrease in the pH-value. At an internal pH of 6.0 inside the proteoliposomes, P_i_ and PEP were even transported with the same rates. The strong pH-dependency of PEP transport could be confirmed in the proteoliposome system with extracts from Col-0 leaves, containing a mixture of all PTs, suggesting that an enhanced PEP transport at pH-values below 8.0 appears to be a general property of the respective PTs. From our data it appears very likely that the 2-fold rather than the 3-fold negatively charged PEP species (i.e., PEP^2-^) is the true transport substrate. Thus the XPT and probably also the PPTs (or GPTs) resemble the TPT with respect to its transport characteristics of phosphorylated organic acids (Fliege et al., 1978). In contrast to 3-PGA or P_i_, which possess p*K*_a_-values for the conversion of the 2^-^ to the 3^-^ forms of 7.1 and 12.5, respectively, the pK_a3_ for PEP lies at around 6.4, indicating that with increasing pH less PEP^2-^ would be available for transport. In particular if the stromal pH of illuminated chloroplasts of about 8.0 is considered, PEP transport into chloroplast from the cytosol at pH-values close to neutral might be irreversible. However, in darkened chloroplasts or non-green plastids, PEP^2-^ transport across the inner envelope might occur in both directions because the steep pH-gradient between both compartments is missing (see also Staehr et al., 2014). PEP transport determined with the recombinant XPT expressed in yeast or PTs extracted from Col-0 leaves depend most likely directly on the availability of PEP^2-^. Thus the pH-dependency of PEP transport is governed by PEP^2-^ concentrations and the *K*_m_-value for PEP^2^, rather than by functional groups of amino acids at the substrate binding site of the PTs. Moreover, differences in inhibition curves of homologous ^32^Pi/^31^Pi exchange by increasing PEP concentrations at pH 7 and 8 might point at altered binding mechanisms. An observation that needs to by addressed in future studies.

## Conclusion and Outlook

Apart from the important experimental proof that the XPT is indeed involved in photoassimilate export from the chloroplast, the data in the present report show a number of new aspects, in particular with reference to PEP transport, that are awaited to be addressed in future studies. The functional role of PPT2 is still not clear. According to **Table 1**, the *A. thaliana* PPT2 has even a higher PEP transport capacity compared to PPT1. Still both single mutant alleles show only a moderate growth phenotype. Moreover, in *cue1-6/ppt-2* double mutants the growth retardation observed for both single mutants was not additive; it was thus governed by the harsh phenotype of *cue1*. More refined analyses are required to elucidate the functional role of PPT2 in wild-type plants as well as in the *cue1-6* background. In addition the physiological role of the XPT as PEP transporter besides of PPT1 is questionable.

Strikingly, there are indications that the GPTs might also be involved in PEP transport. In leaves a functional GPT1 might be responsible for the residual PEP transport observed in extracts of the *cue1-6/ppt2-1/xpt-1* triple mutant (**Figure 7B**). Moreover, it is tempting to speculate that PPT1 and GPT1 might be the only functional PEP transporters in leaves, regardless of *PPT2* expression in the leaf blade (Knappe et al., 2003). If PPT1 is knocked out in *cue1*, the mutant plants show severe growth retardation and the characteristic reticulate leaf phenotype. The intact areas of the leaves, i.e., the bundle sheath, represents the area of all parts of the leaf where GPT1 is most likely functionally expressed, suggesting that the absence of both PTs might worsen the *cue1* phenotype, an assumption that needs to be addressed experimentally. However, previous *PPT1* promoter::reporter gene studies temper the enthusiasm about the above hypothesis, since the *PPT1* promoter is also highly active in the vasculature of the leaves (Knappe et al., 2003). In future studies additional techniques for the verification of tissue or cell type specific expression profiles, like post-translational fusions with reporter genes are required. Moreover, expression profiles of PTs and selected genes involved in metabolic pathways linked by the individual PTs in wild-type and mutant plants of the present report combined with *in silico* studies might help to understand the fine tuning of transcriptional regulation.

Although in recent studies the structural basis for substrate specificities of PTs has been addressed (Lee et al., 2017), the data in the present study indicate that our knowledge on transport characteristics of PTs is still rather sparse. For instance, kinetic constants like *K*_m_ or *K*_i_ were determined only for PTs from certain plant species. Against the background of substantial differences in maximum transport capacities and substrate specificities of PTs from different sources or even from the same source, like the *A. thaliana* PTs, (**Table 1**), this might also be true for their kinetic constants. A detailed and systematical analysis of transport characteristics including pH-dependencies of PTs from one species (in this case *A. thaliana*) is still missing. The knowledge of kinetic constants and substrate specificities of the transporters combined with their temporal and spatial expression pattern is inevitable on the way to model the behavior of plants in response to environmental changes as well as abiotic or biotic stresses. Moreover, local pH-gradients generated for instance by ATPases at the inner envelope ought to be considered as well when transport processes across membranes are evaluated (McCarty et al., 1984). One of the major conclusions, which can be derived from our study, is that our knowledge of the precise roles of certain transporters, here the XPT and PPT2, is still rather fragmentary although interesting and new aspects have been elucidated.

## Author Contributions

EH conducted most of the experiments in the lab. PS isolated the *ppt2-1* mutant. U-IF provided valuable advices and co-supervised the project. RH supervised the project, conducted the transport measurements, and wrote the article.

## Conflict of Interest Statement

The authors declare that the research was conducted in the absence of any commercial or financial relationships that could be construed as a potential conflict of interest.

## Footnotes

1 http://bar.utoronto.ca/efp/cgi-bin/efpWeb.cgi

2 https://www.genomforschung.uni-bielefeld.de/en/projects/former-projects/gabi-till/gabi-till-project/arabidopsis

3 https://imagej.nih.gov/ij/

4 https://www.sigmaaldrich.com/technical-documents/protocols/biology/yeast-transformation-protocols.html

5 https://www.chem.wisc.edu/areas/reich/pkatable/pKa_compilation-1-Williams.pdf

## Supplementary Material

DATA SHEET S1 Contains 8 Supplementary Figures.

DATA SHEET S2 Contains 4 Supplementary Tables including 3 Tables with a statistical analysis.
