# Supplementary material for: The Xylulose 5-Phosphate/Phosphate Translocator Supports Triose Phosphate, but Not Phosphoenolpyruvate Transport Across the Inner Envelope Membrane of Plastids in Arabidopsis thaliana Mutant Plants
Source: Front Plant Sci. 2018 Oct 18;9:1461. doi: 10.3389/fpls.2018.01461 (PMC6201195; doi:10.3389/fpls.2018.01461)
Supplement: DATA SHEET S1 — Contains 8 Supplementary Figures. [file Data_Sheet_1.pdf]

Supplementary Figures

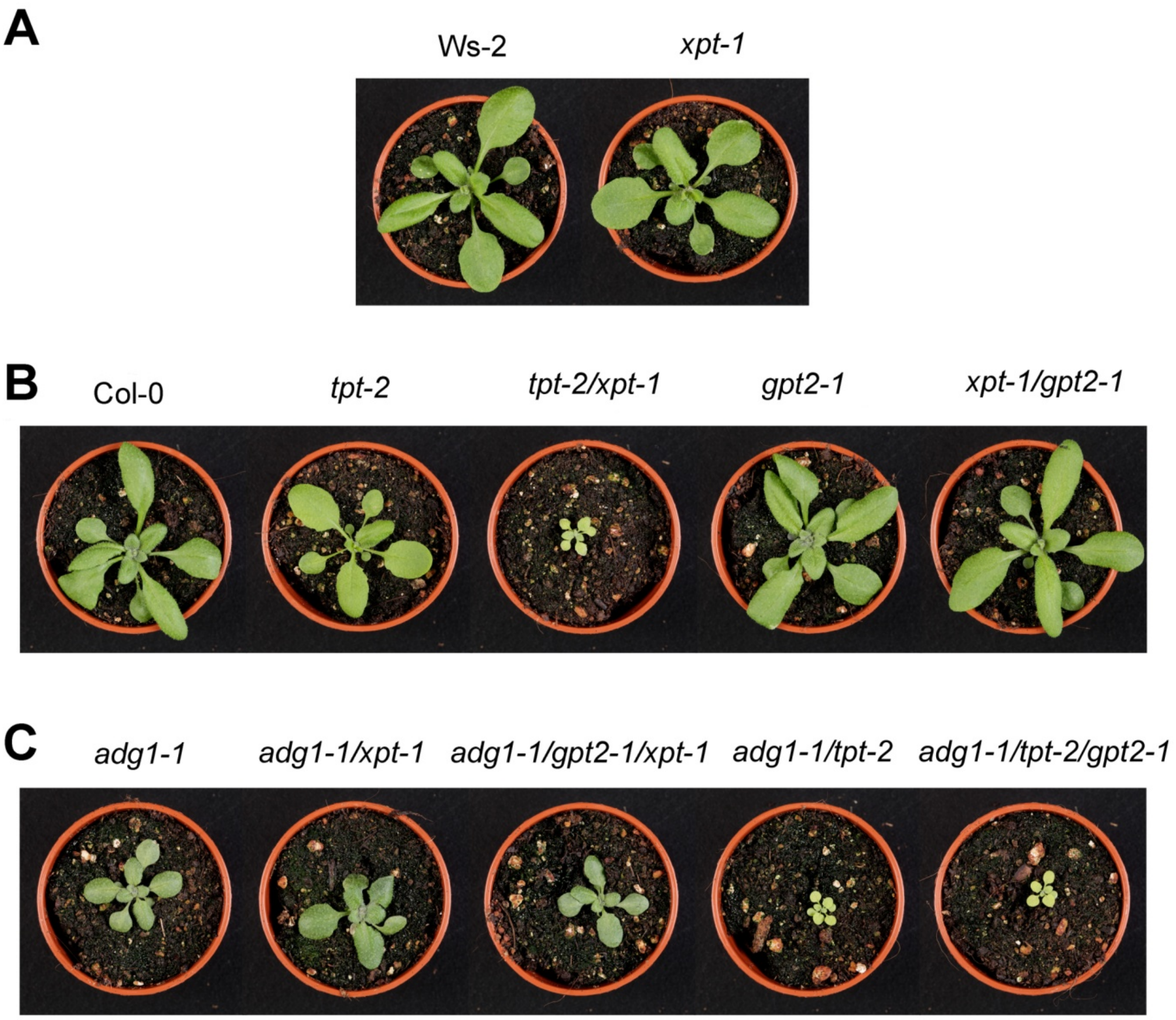

Supplementary Figure 1. Leaf rosettes of wild-type and mutant plants involved in the present study.
Plants were grown for three weeks at a PFD of 150 µmol·m^{-2}·s^{-1} in the long-day. In (**A**) the *xpt-1* single mutant is compared to its wild-type Ws-2. In (**B**) a series of mutants is shown with individual defects in the TPT or GPT2 or in combination with a knockout of the XPT. Col-0 is the background of all other single mutants apart from *xpt-1*. In (**C**) double or triple mutants in the starch-free background *adg1-1* are shown.

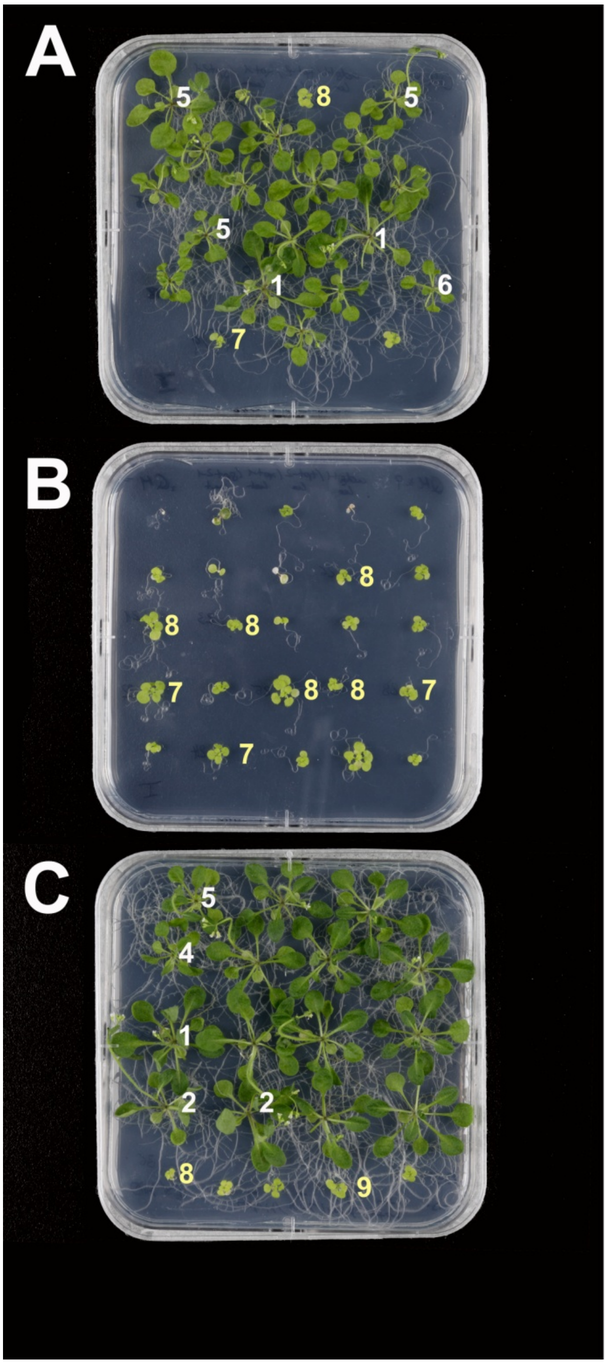

Genotypes

	ADG1	*TPT*	*XPT*	*GPT2*
1	-/-	-/-	+/+	+/+
2	-/-	-/-	+/+	+/-
3	-/-	-/-	+/+	-/-
4	-/-	-/-	+/-	+/+
5	-/-	-/-	+/-	+/-
6	-/-	-/-	+/-	-/-
7	-/-	-/-	-/-	+/+
8	-/-	-/-	-/-	+/-
9	-/-	-/-	-/-	-/-

Supplementary Figure 2. Segregating lines with mutations in the genes *ADG1*, *TPT*, *XPT*, and *GPT2*.

Seeds of a segregating quadruple mutant homozygous for *adg1-1* and *tpt-2*, but heterozygous for *xpt-1* and *gpt2-1* were germinated on 1/2 MS agar plates supplemented with 50 mM sucrose. After nine days the seedlings were analysed for changes in their F_v/F_m-ratio (Figure 1, main manuscript). The seedlings were transplanted to fresh agar plates either randomly (**A** and **C**) or based on the lowest F_v/F_m-ratio (**B**). After 28 days the plants were genotyped and eight possible combinations found. The numbers beside of the plantlets correspondent with the individual genotype on the right panel. Plants with a homozygous defect in *ADG1*, *TPT*, and *XPT* (7 to 9) were extremely growth retarded, irrespectively of the zygosity of *GPT2.*

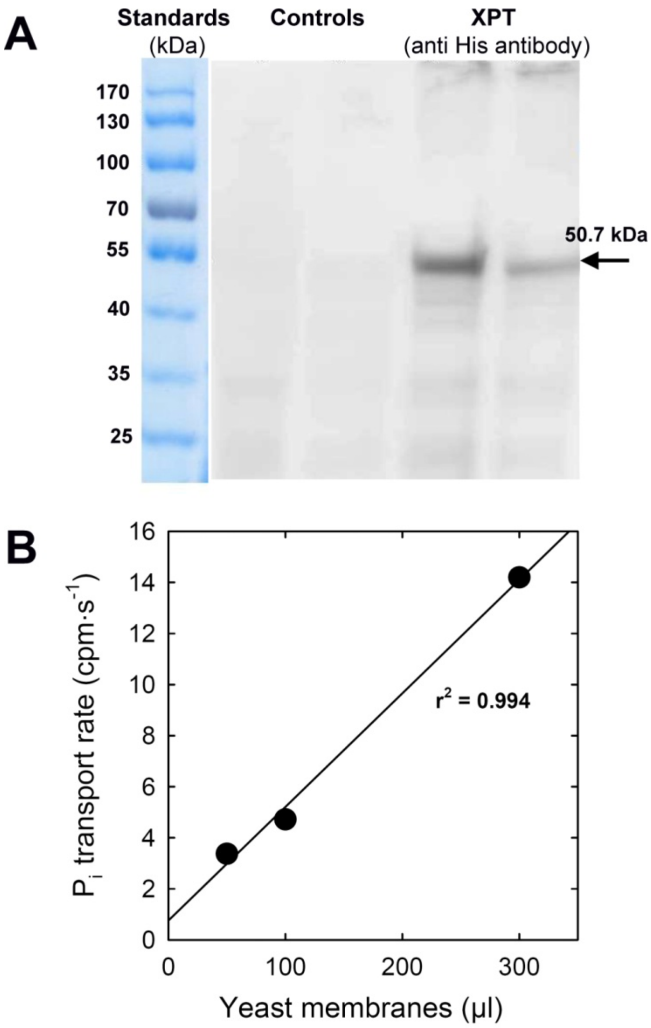

Supplementary Figure 3. Heterologous expression of the *A. thaliana* XPT in yeast.
(**A**) Immunoblots of isolated yeast membranes after induction of XPT expression compared to non-induced controls. (**B**) Dependency of $^{32}P_i/^{31}P_i$ counter exchange rates from the volume of membranes isolated from XPT expressing yeast cells.

A

MFALTFLNPNPRLPSPLFLAKSTPESALSRRSRAFSSSNSYPWRPNLRFNGFKLKSATVPENVEGGDLESGSLVKGLKLGGMFGVWYLLNIYYNIFNKQVLRVY
PYPATVTAFQLGCGTLMIAIMWLLKLHPRPKFSPSQFTVIVQLAVAHTLGNLLTNVSLGRVNVSFTHTIKAMEPFFTVLLSVLLLGEWPSLWIVCSLLPIVAGVSLAS
FTEASFNWIGFCSAMASNVTNQSRNVLSKKFMVGKDALDNINLFSIITIISFILLVPLAILIDGFKVTPSHLQVATSQGLSVKEFCIMSLLAGVCLHSYQQVSYMILEM
VSPVTHSVGNCVKRVVVITSSILFFKTPVSPLNSIGTATALAGVYLYSRAKRVQVKPNPKMS**Stop**

B

MFALTFLNPNPRLPSPLFLAKSTPESALSRRSRAFSSSNSYPWRPNLRFNGFKLKSATVPENVEGGDLESGSLVKGLKLGGMFGVWYLLNIYYNIFNKQVLRVY
PYPATVTAFQLGCGTLMIAIMWLLKLHPRPKFSPSQVNQILIPSNPKNREIHSIVILSSLR**Stop**SYN**Stop**Q**Stop**LTR**Stop**GTC**Stop**QM**Stop**A**Stop**EE**Stop**MSLS
PTLSKQWSLSSPSCFLFSCSVSGRVCGLFVRCYLLSLESL**Stop**HLSQKLLSIGLVSAVQWLLM**Stop**RINHAMSSVKNSWLERTLWTTSTFSL**StopStop**PLSPLS
YWFL**Stop**QSSSMGSRLLLRIYK**Stop**QQARVCR**Stop**KSFASCLYLLVFACIATNRYRI**Stop**YWRWCLQ**Stop**HTL**Stop**GTA**Stop**SVWWLLHHPFFSSKLQSHLL
TL**Stop**VPRLH**Stop**LEYICTAEPNESKSNQIQKCL

C

MFALTFLNPNPRLPSPLFLAKSTPESALSRRSRAFSSSNSYPWRPNLRFNGFKLKSATVPENVEGGDLESGSLVKGLKLGGMFGVWYLLNIYYNIFNKQVLRVY
PYPATVTAFQLGCGTLMIAIMWLLKLHPRPKFSPSQQ**Stop**LTR**Stop**GTC**Stop**Q**Stop**A**Stop**EE**Stop**MSLSPTLSKQWSLSSPSCFLFSCSVSGRVCGLFVRCY
LLSLESL**Stop**HLSQKLLSIGLVSAVQWLLM**Stop**RINHAMSSVKNSWLERTLWTTSTFSL**Stop**PLSPLSYWFL**Stop**QSSSMGSRLLLRIYK**Stop**QQARVCR**Stop**
KSFASCLYLLVFACIATNRYRI**Stop**YWRWCLQ**Stop**HTL**Stop**GTA**Stop**SVWWLLHHPFFSSKLQSHLLTL**Stop**VPRLH**Stop**LEYICTAEPNESKSNQIQKCL

Supplementary Figure 4. Derived amino acid sequences of PPT2 in wild-type and *ppt2-1* plants.
(**A**) Unmodified PPT2 protein of *A. thaliana*. (**B**) Amino acid sequence after incorrect splicing at the mutated site in *ppt2-1* (*ppt2*-tilling-115). (**C**) Amino acid sequence of the PPT2 protein resulting after alternative splicing at position 22 bp downstream of the mutated splice site in *ppt2-1*. The first translational stop is highlighted in grey.

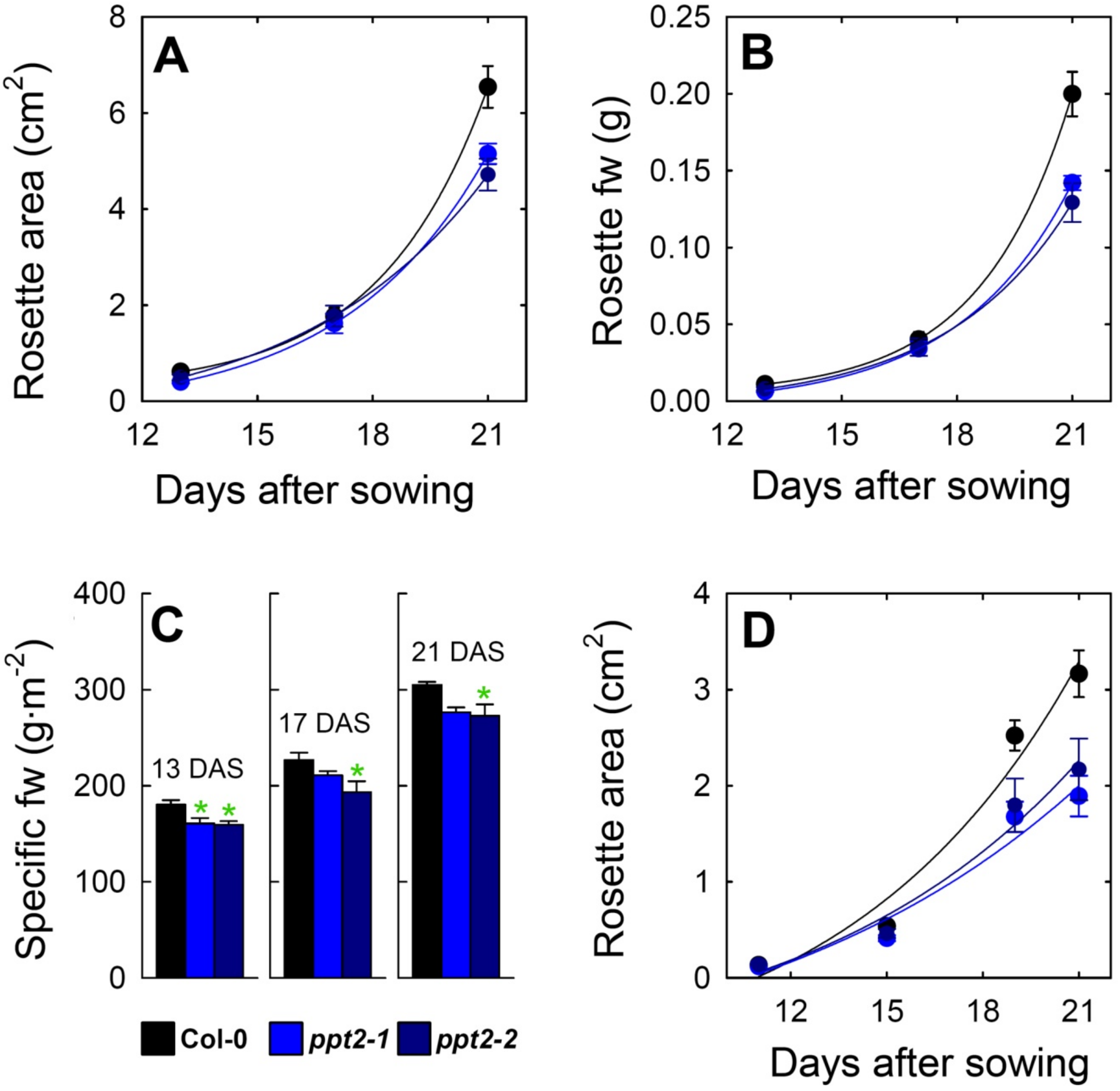

Supplementary Figure 5. Growth characteristics of leaf rosettes of wild-type and *ppt2* mutant plants.

Growth of leaf rosettes were monitored for Col-0 wild-type plants and the *ppt2-1* and *ppt2-2* mutants grown in soil (**A-C**) or on 1/2 MS agar plates (**D**) at a PFD of 150 µmol·m^{-2}·s^{-1} in the long-day. The time dependent increase in either rosette area (**A**) or rosette weight (**B**) was used to calculate specific fresh weights (i.e. fresh weights of the leaves per leaf area) of wild-type and mutant plants (**C**). In (**A**, **B**, and **D**) the closed circles indicate Col-0 (black), *ppt2-1* (light blue), and *ppt2-2* (dark blue). The data represent the mean ± SE of the mean of n = 5 replicates. In (C) significant differences compared to the wild type are indicated by green stars. A statistical analysis of the data is contained in Supplementary Table 2A

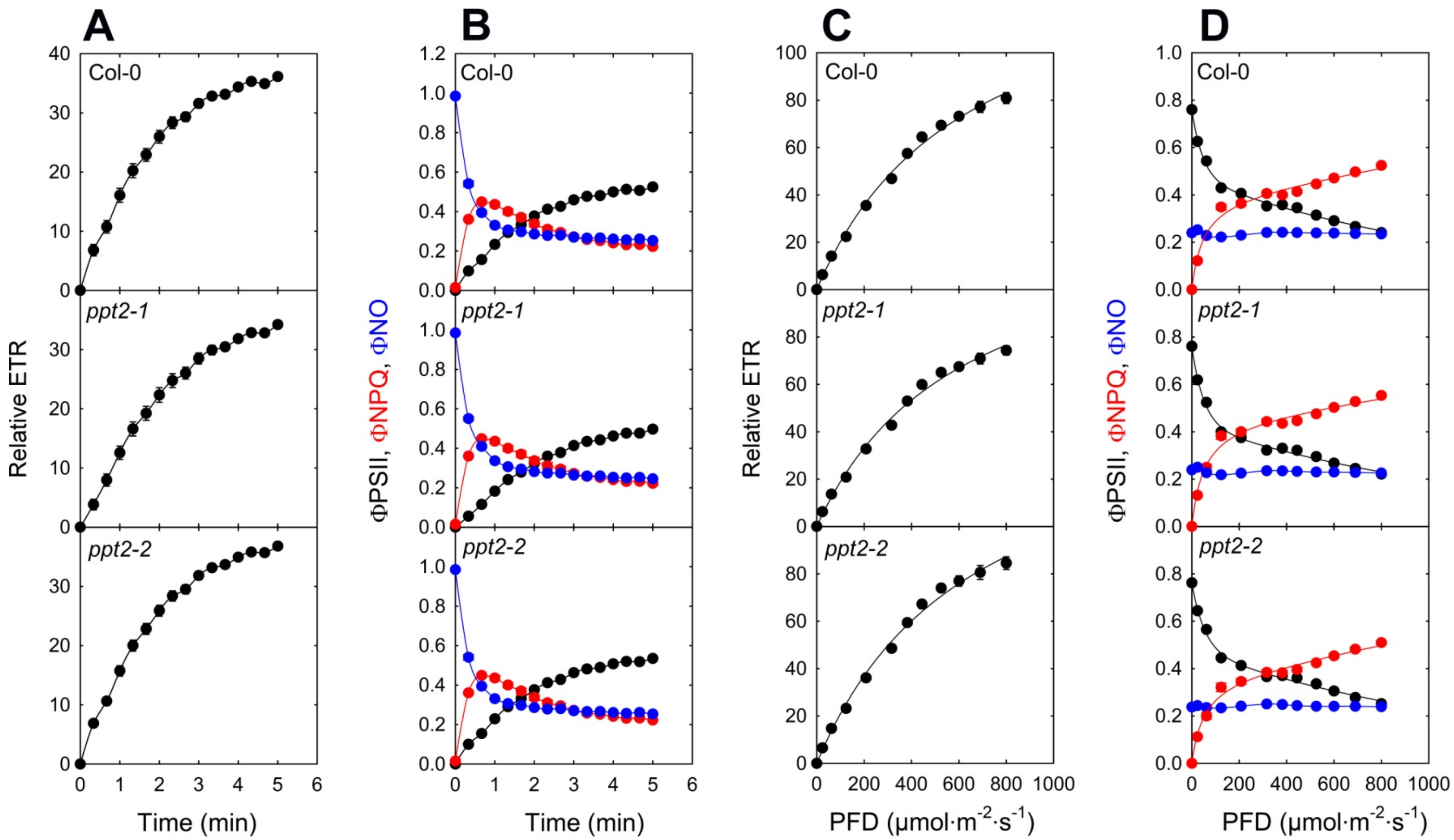

Supplementary Figure 6. Induction and light dependency of photosynthesis in wild-type and *ppt2* mutants.
Plants grown in soil for three weeks at a PFD of 150 µmol·m^{-2}·s^{-1} in the long-day were dark-adapted for 30 min and photosynthesis induced with an actinic PFD of 164 µmol·$^{-2}$·s^{-1}. Relative ETR (**A**) and the efficiencies of PSII and non-photochemical components, i.e. ΦPSII, ΦNPQ, ΦNO (**B**) were monitored during the induction. Shortly after the induction, light curves of relative ETR (**C**), ΦPSII, and NPQ components (**D**) were acquired. The data represent the mean ± SE of the mean with n = 15 replicates. Note that most error bars are smaller than the symbol size. A statistical analysis of the data is contained in Supplementary Table 2B

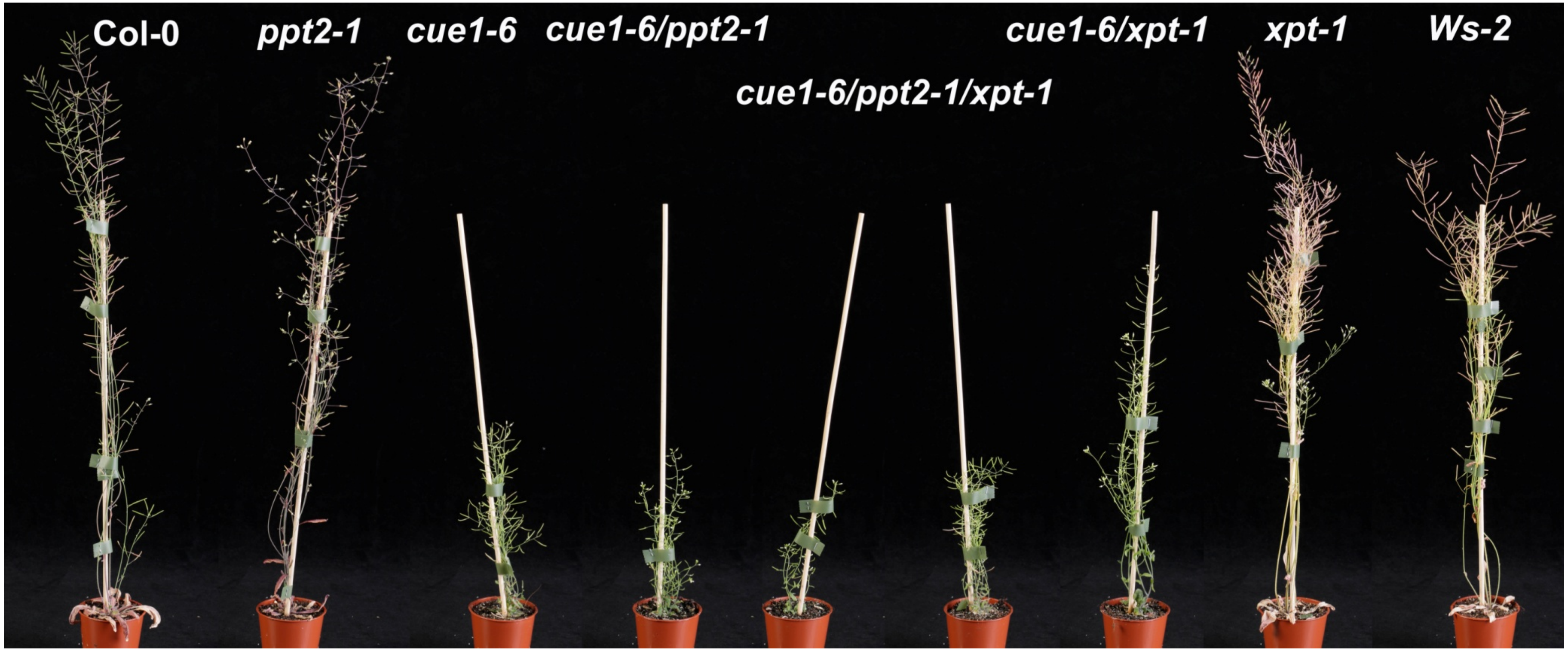

Supplementary Figure 7. Final heights of wild-type and mutant plants impaired in the PPT and/or the XPT.
Plants were grown for 49 days after sowing in soil at a PFD of 150 $\mu mol \cdot m^{-2} \cdot s^{-1}$ in the long-day. The average shoot height is given Table 3 (main article).

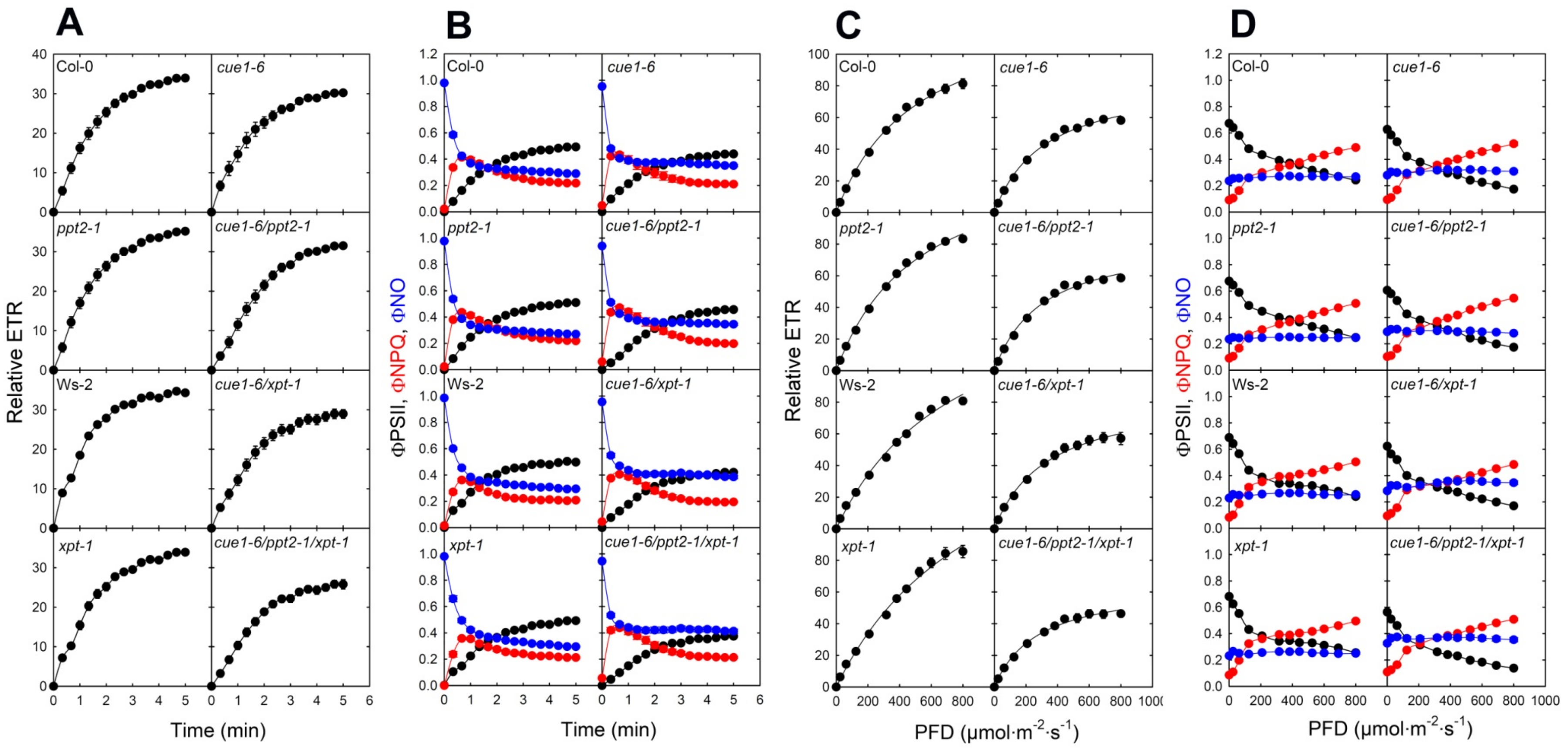

Supplementary Figure 8. Induction and light curves of photosynthesis in wild-type and mutant plants.
Plants grown in soil for three weeks at a PFD of 150 µmol·m^{-2}·s^{-1} in the long-day were dark-adapted for 30 min and photosynthesis induced with an actinic PFD of 164 µmol·$^{-2}$·s^{-1}. Relative ETR (**A**) and the efficiencies of PSII and non-photochemical components, i.e. ΦPSII, ΦNPQ, ΦNO (**B**) were monitored during the induction. Shortly after the induction, light curves of relative ETR (**C**), ΦPSII, and NPQ components (**D**) were acquired. The data represent the mean ± SE of the mean of n = 9 replicates. Note that most error bars are smaller than the symbol size. A statistical analysis of the data is contained in Supplementary Table 4B.
