# Supplementary material for: The Xylulose 5-Phosphate/Phosphate Translocator Supports Triose Phosphate, but Not Phosphoenolpyruvate Transport Across the Inner Envelope Membrane of Plastids in Arabidopsis thaliana Mutant Plants
Source: Front Plant Sci. 2018 Oct 18;9:1461. doi: 10.3389/fpls.2018.01461 (PMC6201195; doi:10.3389/fpls.2018.01461)
Supplement: DATA SHEET S2 — Contains 4 Supplementary Tables including 3 Tables with a statistical analysis. [file Data_Sheet_2.PDF]

Supplementary Tables

Supplementary Table 1. Oligonucleotide primers used for mutant screening and verification. The sequences are given from the 5´ to 3´ end.

A Screening

	***gpt2-1* mutant**
GPT2-1 (f)	GTCGGACCAAACTTTGTCTGGT
GPT2-1 (r)	GGTCTGATCAAGAAATGACACTGA
LB_{gpt2-1} (f)	ATATTGACCATCATACTCATTGC
	***tpt-2* mutant**
TPT-2 (f)	GTAACTTACGAGTAAACTGGCTAC
TPT-2 B (f)	GACCATTAACCCTACCATAACTCC
TPT-2 (r)	TGACTAGCCATGGATACTTGGCGAGGA
LB_{tpt-2} (f)	GTCCGCAATGTGTTATTAAGTTGTC
	***xpt-1* mutant**
XPT-1 (f)	GGCTTTCACCGATTCCCAA
XPT-1 (r)	ATCAAGTAGACGAGGTCAAGAACTAAGTA
LB_{xpt-1} (f)	CATTTTATAATAACGCTGCGGACATCTAC
	***ppt2-1* mutant**
PPT2-1 (f1)	GCCAAGTCTACTCCTGAA
PPT2-1 (r1)	CTAAAGAGACTCCAGCGA
PPT2-1 (f2)	GGGTTTATCCATATCCAGCGAC
PPT2-1 (r2)	GCAAGAGAACAGAAAGCAAGACGG
	***ppt2-2* mutant**
PPT2-2 (f)	GCAGTAGCTCACACGTTAGGG
PPT2-2 (r)	AATAACCACCACACGCTTCAC
LB_{ppt2-2} (f) = LB_{tpt-2} (f)	

B RT-PCR

RT_*PPT2*(f)*	*CACC* ATGTTCGCTCTCACATTTCTAAATCC
RT_*PPT2*(r)	AGACATTTTTGGATTTGGTTTGACTTGGACTCG
RT_*Actin2* (f)	TTGGTAGGCCAAGACATCAT
RT_*Actin2* (r)	GGAGCCTCGGTAAGAAGAAC

*The same primer was or can be used for TOPO cloning

C Yeast expression

XPT TOPO (f)	*CACC* ATGATCTCCCTGAATCTATCTCCT
XPT (r)	GTTCTTCTTATCACCTCCCACTTCAATC

Supplementary Table 2. Statistical analysis (ANOVA/Tukey-Kramer) of growth parameters of wild-type and mutant plants. (**A**) Growth rates of leaf rosettes were determined for plants grown in soil at a PFD of 150 µmol·m^{-2}·s^{-1} in the long-day (Supplementary Figure 6A and B). (**B**) For the determination of photosynthesis parameters three week old soil-grown plants were dark-adapted for 30 min. Photosynthesis was induced with actinic light at a PFD of 164 µmol·m^{-2}·s^{-1}. Relative ETR$_{(800)}$ was determined from light curves at a PFD of 800 µmol·m^{-2}·s^{-1} (Table 2, main article; Supplementary Figure 6). The biotypes are denoted, ***a*** = Col-0, ***b*** = *ppt2-1*, and ***c*** = *ppt2-2.* The significance levels of P < 0.05 or P < 0.01 are indicated by light or dark blue colors.

A

3 comparisons

**Growth characteristics**	***b vs a***	***c vs a***	***b vs c***
**Rosette area**			
13 DAS			
17 DAS			
21 DAS			
**Rosette fw**			
13 DAS			
17 DAS			
21 DAS			
**Rosette specific fw**			
13 DAS			
17 DAS			
21 DAS			

B

**Chl *a* fluorescence parameters**	***b vs a***	***c vs a***	***b vs c***
**PAM**			
F_v/F_m-ratio			
$\Phi PSII_{(164)}$			
$\Phi NPQ_{(800)}$			
$\Phi NO_{(800)}$			
Relative ETR$_{(800)}$			

Supplementary Table 3. Statistical analysis (ANOVA/Tukey-Kramer) of growth, photosynthesis, and leaf parameters in wild-type and mutant plants. (A) Growth parameters of rosette leaves were assessed of plants grown in soil under long-day conditions at a PFD of 150 µmol·m^{-2}·s^{-1} (see Figure 6D, main article). Generative growth parameters are contained in Table 3 (main article). Chl fluorescence parameters (**B**) and leaf composition (**C**) contained in Table 4 (**A** and **B**; main article) and for non-photochemical quench parameters in Supplementary Figure 8. The biotypes are denoted, ***a*** = Col-0, ***b*** = Ws-2, ***c*** = *xpt-1*, ***d*** = *cue1-6*, ***e*** = *ppt2-1*, ***f*** = *cue1-6/ppt2-1*, ***g*** = *cue1-6/xpt*-1, and ***h*** = *cue1-6/ppt2-1/xpt-1*. DAS = days after sowing. The significance levels of P < 0.05 or P < 0.01 are indicated by light or dark blue colors.

A
8 comparisons

	b vs a	*c vs a*	*d vs a*	*e vs a*	*f vs a*	*g vs a*	*h vs a*	*c vs b*	*d vs b*	*e vs b*	*f vs b*	*g vs b*	*h vs b*	*d vs c*	*e vs c*	*f vs c*	*g vs c*	*h vs c*	*e vs d*	*f vs d*	*g vs d*	*h vs d*	*f vs e*	*g vs e*	*h vs e*	*g vs f*	*h vs f*	*h vs g*
**Rosette area**																												
11 DAS	dark		dark	dark	dark	dark	dark	dark	dark		dark	dark	dark	dark	light	dark	dark	dark	dark				dark	dark	dark			
15 DAS	dark	light	dark	dark	dark	dark	dark	dark	dark		dark	dark	dark	dark	dark	dark	dark	dark	dark				dark	dark	dark			
19 DAS	dark	dark	dark	dark	dark	dark	dark		dark	dark	dark	dark	dark	dark	dark	dark	dark	dark	dark				dark	dark	dark			
22 DAS	dark	dark	dark	dark	dark	dark	dark		dark	dark	dark	dark	dark	dark	dark	dark	dark	dark	dark				dark	dark	dark			
**Generative growth**																												
Final height			dark		dark	dark	dark		dark		dark	dark	dark	dark	dark	dark	dark	dark	dark		dark		dark	dark	dark			
Silique number	dark	dark	dark		dark	dark	dark	light	dark	dark	dark	dark	dark	dark	dark	dark	dark	dark	dark				dark	dark	dark			

Supplementary Table 3 (continued)

B

**Chl *a* fluorescence parameters**	***b vs a***	***c vs a***	***d vs a***	***e vs a***	***f vs a***	***g vs a***	***h vs a***	***c vs b***	***d vs b***	***e vs b***	***f vs b***	***g vs b***	***h vs b***	***d vs c***	***e vs c***	***f vs c***	***g vs c***	***h vs c***	***e vs d***	***f vs d***	***g vs d***	***h vs d***	***f vs e***	***g vs e***	***h vs e***	***g vs f***	***h vs f***	***h vs g***
F_v/F_m																												
$\Phi PSII_{(164)}$																												
$\Phi NPQ_{(800)}$																												
$\Phi NO_{(800)}$																												
Relative $ETR_{(800)}$																												

C

**Leaf parameters**	***b vs a***	***c vs a***	***d vs a***	***e vs a***	***f vs a***	***g vs a***	***h vs a***	***c vs b***	***d vs b***	***e vs b***	***f vs b***	***g vs b***	***h vs b***	***d vs c***	***e vs c***	***f vs c***	***g vs c***	***h vs c***	***e vs d***	***f vs d***	***g vs d***	***h vs d***	***f vs e***	***g vs e***	***h vs e***	***g vs f***	***h vs f***	***h vs g***
Chlorophyll																												
Carotenoids																												
Chl *a*/*b*-ratio																												
Chl/Car-ratio																												
Protein																												
Spec. leaf fw																												

a = Col-0, ***b*** = Ws-2, ***c*** = *xpt-1*, ***d*** = *cue1-6*, ***e*** = *ppt2-1*, ***f*** = *cue1-6/ppt2-1*, ***g*** = *cue1-6/xpt*-1, and ***h*** = *cue1-6/ppt2-1/xpt-1*.

Supplementary Table 4. Statistical analysis (ANOVA/Tukey-Kramer) of transport rates determined with the proteoliposome system. The transport rates ΔP_i and ΔPEP indicate the unspecific gluconate rate was substracted (see Figure 7, main article). The biotypes are denoted, ***a*** = Col-0, ***b*** = *ppt2-1*, ***c*** = *cue1-6*, ***d*** = *cue1-6/ppt2-1,* and ***e*** = *cue1-6/ppt2-1/xpt-1*. The significance levels of $P < 0.05$ or $P < 0.01$ are indicated by light or dark blue colors.

5 comparisons

**Transport rates**	***b vs a***	***c vs a***	***d vs a***	***e vs a***	***c vs b***	***d vs b***	***e vs b***	***d vs c***	***e vs c***	***e vs d***
ΔP_i										
ΔPEP										
